# Activation of RAS/MAPK pathway confers MCL-1 mediated acquired resistance to BCL-2 inhibitor venetoclax in acute myeloid leukemia

**DOI:** 10.1038/s41392-021-00870-3

**Published:** 2022-02-21

**Authors:** Qi Zhang, Bridget Riley-Gillis, Lina Han, Yannan Jia, Alessia Lodi, Haijiao Zhang, Saravanan Ganesan, Rongqing Pan, Sergej N. Konoplev, Shannon R. Sweeney, Jeremy A. Ryan, Yulia Jitkova, Kenneth Dunner, Shaun E. Grosskurth, Priyanka Vijay, Sujana Ghosh, Charles Lu, Wencai Ma, Stephen Kurtz, Vivian R. Ruvolo, Helen Ma, Connie C. Weng, Cassandra L. Ramage, Natalia Baran, Ce Shi, Tianyu Cai, Richard Eric Davis, Venkata L. Battula, Yingchang Mi, Jing Wang, Courtney D. DiNardo, Michael Andreeff, Jeffery W. Tyner, Aaron Schimmer, Anthony Letai, Rose Ann Padua, Carlos E. Bueso-Ramos, Stefano Tiziani, Joel Leverson, Relja Popovic, Marina Konopleva

**Affiliations:** 1 Department of Leukemia, The University of Texas MD Anderson Cancer Center, Houston, TX USA; 2 AbbVie Inc., North Chicago, IL USA; 3 Institute of Hematology, Chinese Academy of Medical Sciences & Peking Union Medical College, Tianjin, China; 4 Department of Nutritional Sciences, Department of Pediatrics, Department of Oncology, Dell Medical School, The University of Texas at Austin, Austin, TX 78712 USA; 5 Department of Cell, Developmental & Cancer Biology, Division of Hematology & Medical Oncology, Knight Cancer Institute, Oregon Health & Science University, Portland, OR USA; 6 Université de Paris, Institut de la Recherche Saint-Louis (IRSL), Inserm Unit 1131, Paris, France; 7 Dana-Farber Cancer Institute, Boston, MA USA; 8 Department of Hematopathology, The University of Texas MD Anderson Cancer Center, Houston, TX USA; 9 Princess Margaret Cancer Center, Toronto, ON Canada; 10 High Resolution Electron Microscopy Facility, The University of Texas MD Anderson Cancer Center, Houston, TX USA; 11 Department of Bioinformatics & Computational Biology, The University of Texas MD Anderson Cancer Center, Houston, TX USA; 12 Department of Hematology, The First Hospital Affiliated Harbin Medical University, Harbin, China; 13 Department of Lymphoma & Myeloma Research, The University of Texas MD Anderson Cancer Center, Houston, TX USA

**Keywords:** Haematological cancer, Translational research

## Abstract

Despite high initial response rates, acute myeloid leukemia (AML) treated with the BCL-2–selective inhibitor venetoclax (VEN) alone or in combinations commonly acquires resistance. We performed gene/protein expression, metabolomic and methylation analyses of isogenic AML cell lines sensitive or resistant to VEN, and identified the activation of RAS/MAPK pathway, leading to increased stability and higher levels of MCL-1 protein, as a major acquired mechanism of VEN resistance. MCL-1 sustained survival and maintained mitochondrial respiration in VEN-RE cells, which had impaired electron transport chain (ETC) complex II activity, and MCL-1 silencing or pharmacologic inhibition restored VEN sensitivity. In support of the importance of RAS/MAPK activation, we found by single-cell DNA sequencing rapid clonal selection of RAS-mutated clones in AML patients treated with VEN-containing regimens. In summary, these findings establish RAS/MAPK/MCL-1 and mitochondrial fitness as key survival mechanisms of VEN-RE AML and provide the rationale for combinatorial strategies effectively targeting these pathways.

## Introduction

Acute myeloid leukemia (AML) is the most common type of adult leukemia. With chemotherapy and hematopoietic stem cell transplantation, only approximately 30% of patients with non-acute-promyelocytic leukemia (APL) forms of AML survive for more than 5 years. Nearly half of young patients and up to 80% of elderly patients die of relapsed or refractory disease or treatment-related complications.^1^ The recent introduction of BCL-2 inhibitor venetoclax (VEN) in combination with low dose chemotherapy or hypomethylating agents achieved high response rates even in patients traditionally thought to be unfit for standard chemotherapy, and received accelerated Food and Drug Administration (FDA) approval in 2018.^2,3^ However, despite high rates of remissions, the majority of the responses are transient and culminate in chemorefractory relapse.^4^ Therefore, identifying and targeting mechanisms of VEN resistance remains critically important.

BCL-2 family proteins are key regulators of the mitochondrial pathway of apoptosis through regulation of outer mitochondrial membrane integrity. In response to cell death signals, the proapoptotic (“BH3-only”) BCL-2 family members (such as BIM, PUMA, and BID) are released from antiapoptotic members (such as BCL-2, MCL-1) and activate the death effectors BAX and BAK. Activated BAX and BAK then oligomerize and form pores in the mitochondrial outer membrane. This process, known as mitochondrial outer membrane permeabilization, releases cytochrome c into the cytosol, which in turn activates caspase cascade that leads to apoptosis. Several studies have reported intrinsic resistance to VEN, including overexpression of antiapoptotic BCL-2 family members other than BCL-2,^5^ acquired mutations in the BCL-2 binding groove found in chronic lymphocytic leukemia (CLL),^6^ amplifications of *BCL2*,^2,6,7^ and alterations in the mitochondria structure and metabolic changes.^8^

Overexpression of MCL-1 was reported by us and others as one of the key mechanisms of resistance to BCL-2 inhibitors, through sequestering BH3-only proteins released from BCL-2, and preventing activation of BAX/BAK.^5,9^ Lin et al.^10^ reported that increased MCL-1 stability mediates resistance to VEN; however, the mechanisms of MCL-1 upregulation are not known. While the antiapoptotic function of BCL-2 and MCL-1 is well understood, they additionally have secondary functions regulating mitochondrial metabolism that are less recognized. Gene expression profiles of RNA extracted from Sca1^+^ spleen cells of the mutant NRAS/BCL-2 high risk myelodysplastic syndrome (HR-MDS) and the AML post MDS mouse models showed significant upregulation of genes coding for components of the major complexes of the mitochondrial electron transport chain (ETC) including genes of complex I, IV for HR-MDS model and Complex I, II, III, IV, and V for AML post MDS model^11^. BCL-2 family members co-localize on mitochondria with enzymes from various metabolic pathways. For instance, BCL-XL interacts with the voltage-dependent anion channel protein (VDAC) that mediates ADP/ATP exchange across the mitochondrial membrane, and facilitates mitochondrial respiration.^12,13^ BCL-2 was reported to be upregulated in quiescent leukemia stem cells (LSCs) where it maintains oxidative phosphorylation;^14^ in a recent report, the combination of VEN and the hypomethylating agent (HMA) azacitidine disrupted the tricarboxylic acid cycle in LSCs by targeting glutathionylation of mitochondrial complex II of the ETC.^15^ N-truncated MCL-1 is localized to the mitochondrial matrix, where it maintains normal inner mitochondrial membrane (IMM) structure, regulates mitochondrial fusion, and supports mitochondrial bioenergetic functions.^16^ Furthermore, the IMM form of MCL-1 interacts with very long-chain acyl-CoA dehydrogenase, a key enzyme in the mitochondrial fatty acid oxidation pathway.^17^ Importantly, the roles of both BCL-2 and MCL-1 in regulating mitochondrial metabolism are independent of their antiapoptotic function.

In this study, we established AML cell line models with acquired resistance to VEN. We found that venetoclax-resistant (VEN-RE) cells exhibited drastic changes in global DNA methylation and robust activation of the MAPK pathway, leading to upregulation of MCL-1, which in turn sustains mitochondrial respiration and cellular metabolism. Genetic silencing or pharmacological inhibition of MCL-1 restored VEN sensitivity. Single-cell DNA sequencing demonstrated clonal selection of RAS-mutated clones in AML patients treated with VEN-containing regimens, and combined VEN with MCL-1 inhibitor exhibited superior anti-leukemia effect in MOLM-13 gain-of-function NRAS-G12D mutation in vitro and mouse model in vivo, supporting the future need to co-target this signaling axis to improve the longevity of responses.

## Results

### Genomes of VEN-RE AML cells show extensive differential methylation, and HMA partially reverse VEN resistance

**Fig. 1:**
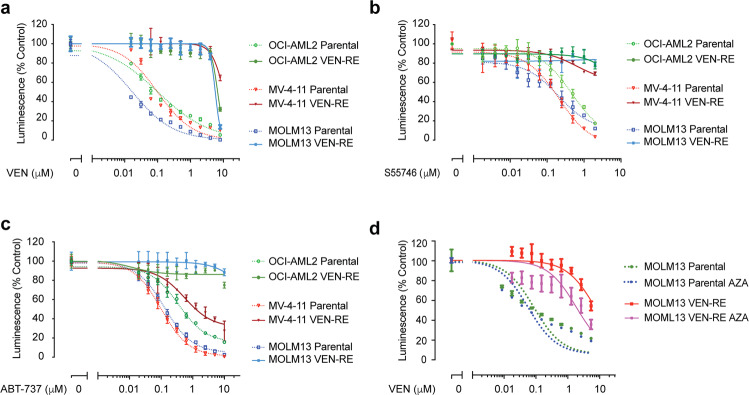
Venetoclax-resistant (VEN-RE) cells had hypermethylated genomes. **a**–**d** The indicated parental cells (dotted lines) and VEN-RE cells (solid lines) were treated with the indicated inhibitors for 48 h, then cell viability was determined by CellTiter-Glo assay. Luminescence reads were normalized to those of DMSO-treated control cells. Data are mean ± SD. **a** Results of viability assays for cells treated with 0–10 μM VEN. **b** Results of viability assays for cells treated with 0–2 μM of the BCL-2 inhibitor S55746. **c** Results of viability assays for cells treated with 0–10 μM of the BCL-2/BCL-XL inhibitor ABT-737. **d** Viability of MOLM-13 parental and VEN-RE exposed to AZA and subsequently treated with 0–10 μM VEN

We first established in vitro acquired resistance models in 3 AML cell lines that were initially sensitive to VEN (OCI-AML2, MV-4-11, and MOLM-13). By exposing the parental cells to gradually increasing concentrations of VEN, we generated “VEN-RE” cells that, after 5 to 8 weeks of selection, ultimately were able to survive exposure to ≥ 1 μM VEN. We next performed single-cell plating and selected single-cell clones from the populations of VEN-RE cells (Fig. 1a; the schema of the selection process is shown in Supplementary Fig. 1a). The VEN-RE cells exhibited a slightly slower growth rate compared to the parental cells (Supplementary Fig. 1b). The IC_50_ of VEN in VEN-RE cells was 21- to 88-fold higher than in the parental cells, with an average of 6.7 µM in VEN-RE cells compared to 0.1 µM in parental cells (Table 1). The withdrawal of VEN for 48 h did not restore sensitivity to the inhibitor (Supplementary Fig. 1c).

**Table 1:** VEN IC_50_ in parental and VEN-RE cells

IC_50_ (µM)			
Cell line	Parental	VEN-RE	Fold change
OCI-AML2	0.150 ± 0.053	3.095 ±0.779	21
MV-4-11	0.138 ± 0.043	13.753 ± 4.422	69
MOLM-13	0.037 ± 0.019	3.263 ± 1.730	88

To determine whether the resistance was general against the class of BCL-2/BCL-XL inhibitors or specific to VEN, we tested the cells’ sensitivity to other BCL-2 inhibitors, including BCL-2 inhibitor S55746 and the dual BCL-2/BCL-XL inhibitor ABT-737 (Fig. 1b, c). The VEN-RE cells exhibited cross-resistance to S55746 and ABT-737, indicating that the VEN-RE cells were generally resistant to BCL-2 inhibition, not only to VEN. We next studied mechanisms of resistance utilizing whole genome sequencing, methylation arrays and RNA sequencing approaches. Since mutations in the BH3 binding groove of BCL-2 were shown to cause a conformational change that confers resistance to VEN in CLL,^7^ we investigated potential emergence of BCL-2 family members mutations in the VEN-RE cells using whole genome sequencing. Comparison of VEN-RE to parental cells did not identify any coding mutations in BCL-2, BAX, or other BCL-2 family members (data not shown). Mutations in genes outside the apoptotic pathway were detected in some of the VEN-RE cells, among which, missense mutation of *OGT* (O-Linked N-Acetylglucosamine (GlcNAc) Transferase) was the only shared mutated gene in VEN-RE MOLM-13 and OCI-AML2 cells (Supplementary Table 1), however, their potential role in VEN resistance is not clear at this time and requires further investigation.

We next compared DNA methylation profiles using Infinium EPIC arrays in paired isogenic parental and VEN-RE cells. Of interest, the genomes of VEN-RE cells exhibited globally altered methylation across various regions. Compared to parental cells, VEN-RE cells (pooled cell lines and single clones) had 4999 differentially methylated sites (range 14,723–36,015) involving 3423 genes (8262–13,292) that had more than 20% difference in methylation and were within 2 kb of the transcription start site (TSS) (with false discovery rate [FDR] 0.1) (Supplementary Table 2). The majority of the differentially methylated genes were shared between at least two cell lines, with 4381 genes having differential methylation surrounding their TSS across all three VEN-RE cell lines (Supplementary Fig. 1d).

To establish correlation of the DNA methylation changes with gene expression, we next performed RNA sequencing of the cells to identify genes that were significantly different in terms of both DNA methylation and RNA expression. The number of concordant genes identified by both assays varied from 104 genes in paired MV-4-11 cells, 753 genes in MOLM-13 cells to 1775 genes in OCI-AML2 cells (Supplementary Fig. 1e). The DNA methylation analysis revealed decreased methylation of several sites within the *MCL-1* promoter in VEN-RE OCI-AML2 and MOLM-13 cells, which correlated with a log2 fold increase of 0.73 by RNAseq differential gene expression analysis (Supplementary Fig. 1f).

We next used the HMA azacitidine to study the impact of DNA demethylation on sensitivity of the VEN-RE cells. We treated the VEN-RE cells with 10 nM azacitidine, which did not induce any cell death, for 4 weeks on a 2 days on/2 days off schedule. The VEN dose-response curve shifted left in VEN-RE cells after completion of azacitidine therapy, demonstrating that HMA treatment at least partially re-sensitized the cells to VEN (Fig. 1d). This increase in sensitivity, but not a full reversal of resistance, indicates that aberrant methylation of the VEN-RE cells’ genomes contributes to VEN resistance, and that additional mechanisms exist.

### VEN-RE cells exhibit increased MCL-1 expression and functional dependence

**Fig. 2:**
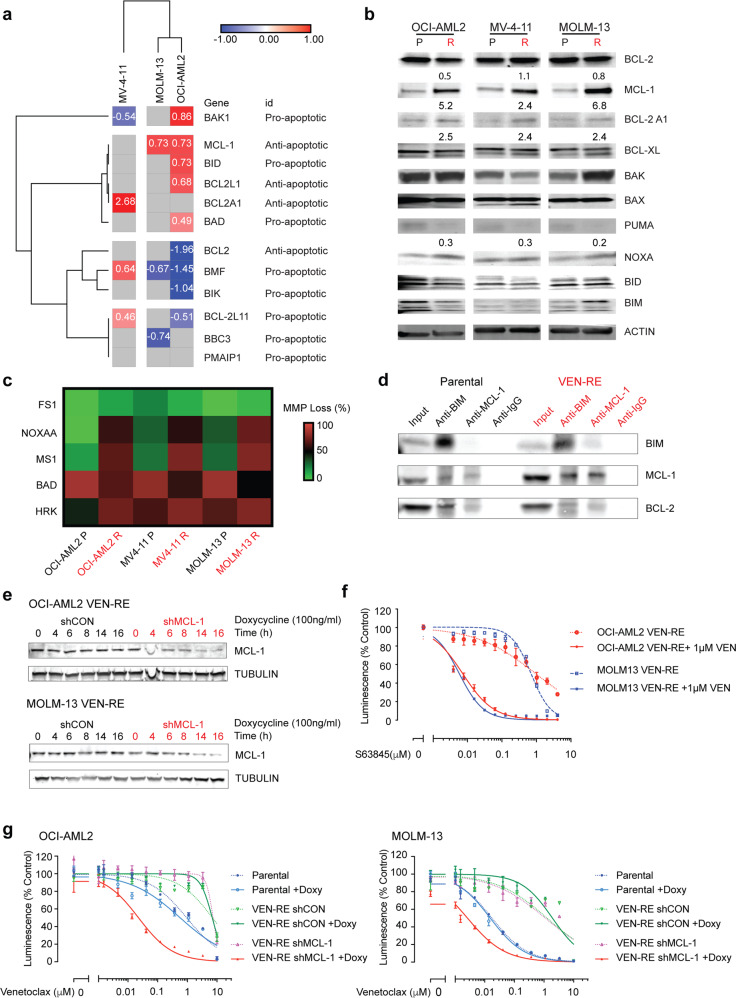
MCL-1 became the primary antiapoptotic protein in VEN-RE cells. **a** Heatmap showing results of RNA sequencing analysis of BCL-2 family genes in parental and VEN-RE AML cell lines (FDR ≤ 0.2). Genes in red had higher expression, and genes in blue had ower expression, in VEN-RE cells. The numbers in the cells represent normalized expression levels to those in parental lines. **b** Western blots showing BCL-2 family protein expression in the indicated parental (P) and VEN-RE (R) cell lines. Numbers below the bands are quantifications normalized to levels in the parental lines. **c** Heatmap showing results of BH3 profiling of the indicated parental and resistant AML cell lines when exposed to the indicated peptides. Data were normalized to DMSO (as negative control) and FCCP (as positive control). The colors indicate the loss of mitochondrial membrane potential (MMP). **d** Co-immunoprecipitation of parental and VEN-RE MOLM-13 cells protein lysates pulled down with anti-BIM and anti-MCL-1 to determine the binding of BIM with BCL-2 and MCL-1. IgG served as a negative control. **e** Western blots showing inducible MCL-1 expression. MCL-1 knockdown was induced with 100 ng/mL doxycycline for 0–8 h in VEN-RE MOLM-13 and for 0–16 h in VEN-RE OCI-AML2 cells transfected with a control shRNA (shCON) or shMCL-1. **f** VEN-RE OCI-AML2 and MOLM-13 cells were treated with 0-4 μM of the MCL-1 inhibitor S63845 with (solid lines) or without 1 μM VEN (dotted lines) for 48 h, then cell viability was determined by CellTiter-Glo assay. Luminescence reads were normalized to those of DMSO-treated controls. Data represent mean ± SD from triplicate independent experiments. **g** Parental, VEN-RE, and VEN-RE OCI-AML2 and MOLM-13 cells transfected with a control shRNA (shCON) or a shRNA targeting MCL-1 (shMCL-1) that were induced with (solid lines) or without 100 ng/mL doxycycline (dotted lines) and treated with 0–10 μM VEN for 48 h. Cell viability was determined by CellTiter-Glo assay. Luminescence reads were normalized to DMSO-treated control cells. Data are mean ± SD

RNA sequencing revealed cell line-specific changes in the expression of BCL-2 family and TNF/TRAIL death receptor pathway genes, which was generally consistent with the shift in dependence on anti- and/or proapoptotic members, yet distinct among different cell lines (Fig. 2a and Supplementary Fig. 2a). In VEN-RE MOLM-13 cells, we found increased expression of the antiapoptotic gene *MCL1* and decreased expression of the proapoptotic genes *BBC3* (coding gene for PUMA) and *BMF*. These changes suggested that instead of depending on BCL-2 for survival, VEN-RE MOLM-13 cells rely on changes in *MCL1* and *BBC3/BMF* expression. In VEN-RE OCI-AML2 cells, we found downregulation of antiapoptotic *BCL2* and upregulation of *MCL1* and *BCL2L1* (BCL-XL); reduced expression of proapoptotic *BMF*, *BIK*, and *BCL2L11* (BIM). And in VEN-RE MV-4-11 cells, there was increased expression of antiapoptotic *BCL2A1* (BFL1) and decreased expression of proapoptotic *BAK1*. Similarly, the TNF/TRAIL death receptor pathway also showed cell line-specific changes involving multiple proapoptotic and antiapoptotic members, such as increased *LMNA* and *APAF1* and decreased *TNFRSF8, TNFSF10, FHIT*, and *RIPK1* expression in VEN-RE MOLM-13 cells (Supplementary Fig. 2a).

We next performed reverse phase protein array (RPPA) analysis of the paired cell lines. The RPPA analysis revealed decreased expression of proapoptotic BCL-2 family protein PUMA and BAX in the VEN-RE lines, increased adhesion proteins P-Cadherin and ROCK-1, decreased PDCD4 expression and upregulation of several proteins indicating activation of the RAS/MAPK pathway, such as increased ELK1 and SHC expression (Supplementary Fig. 2b).

To validate the RNA sequencing and RPPA findings and investigate whether changes in BCL-2 family members at the protein level contributed to VEN resistance, we performed immunoblotting analysis. This confirmed increased MCL-1 and BFL1 expression and decreased levels of the proapoptotic BH3-only protein PUMA in VEN-RE lines (Fig. 2b). Similar to the RNA sequencing results, which showed cell line-specific BCL-2 family changes, no other changes in the BCL-2 family proteins tested were shared among all cell lines. High levels of MCL-1 protein and rather modest increase in MCL-1 mRNA despite DNA hypomethylation suggests that post-transcriptional protein modification(s) contributed to the increased protein level in some cell lines.

To investigate the functional importance of the altered BCL-2 proteins, we next conducted BH3 profiling. In BH3 profiling, cells are exposed to different BH3 mimetic peptides and cytochrome c release is measured; BH3 profiling has proven useful for investigating tumor dependency on selected BCL-2 antiapoptotic members for survival.^18,19^ While we found no difference in overall priming to BIM and PUMA between the parental and VEN-RE cells, we observed increased priming to the peptides NOXA-A, MS1, and HRK in VEN-RE cell lines, indicating increased dependence on antiapoptotic MCL-1 and BCL-XL, both of which are not targeted by VEN (Fig. 2c, Supplementary Fig. 2c, d).

As a BH3 mimetic, VEN displaces the BH3-only protein BIM from BCL-2 to initiate apoptosis in cells with low levels of MCL-1. We examined whether the increased MCL-1 level would affect the BIM binding pattern in VEN-RE cell lines. Co-immunoprecipitation (co-IP) experiments showed reduced pull-down of BCL-2 by BIM in VEN-RE cells and increased levels of BIM binding to MCL-1 (Fig. 2d). This data indicates that MCL-1 sequesters most BIM protein away from BCL-2 in VEN-RE cells. To confirm the antiapoptotic role of MCL-1 in VEN-RE cells, we utilized doxycycline-inducible MCL-1 knockdown^20^ in VEN-RE OCI-AML2 and MOLM-13 cells to silence MCL-1 and determine whether this would re-sensitize VEN-RE cells to VEN. With 100 ng/mL doxycycline, the MCL-1 level was reduced by more than half at 6 h in VEN-RE MOLM-13 cells and at 14 h in VEN-RE OCI-AML2 cells (Fig. 2e). While the cell viability remained unaffected, MCL-1 silencing by shRNA resulted in a full reversal of the resistance to VEN (Fig. 2f). Consistently, a selective MCL-1 inhibitor S63845 re-sensitized the VEN-RE cells to VEN (Fig. 2g). Taken together, our findings suggest that MCL-1 is a dominant antiapoptotic protein in these VEN-RE cells and MCL-1 inhibition re-sensitized the cells to VEN.

### MCL-1 maintains mitochondrial respiration in VEN-RE cells

**Fig. 3:**
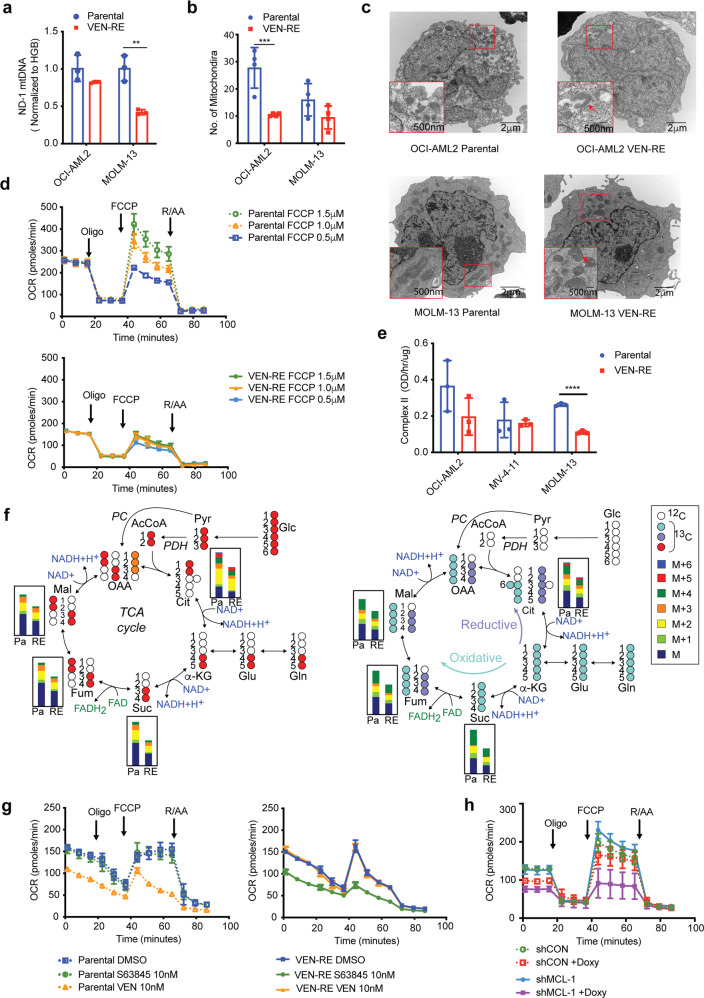
VEN-RE cells were deficient in mitochondrial respiration. **a** Mitochondrial *ND-1* DNA expression levels were detected by RT-PCR and normalized to *HGB* levels in the indicated parental and VEN-RE cells. **P < 0.005. **b** Number of mitochondria in five randomly selected parental and VEN-RE cells in different fields under transmission electron microscopy (TEM). ***P < 0.0005. **c** TEM images of mitochondrial morphology. Areas marked in red are enlarged in the insets on each panel. Scale bars indicate 2 μm in the larger windows and 500 nm in the insets. **d** Results of Seahorse assays showing oxygen consumption rate (OCR) with the indicated concentrations of FCCP) in parental (dotted lines) and VEN-RE MOLM-13 cells (solid lines). **e** Electron transport chain complex II activity in parental and VEN-RE cell lines. ****P < 0.0001. **f** ^13^C labeling pattern of TCA cycle intermediates following labeling with U-^13^C-glucose (left) or ^13^C_5_,^15^N_2_-glutamine (right). Bar plots show the relative intensity of stable isotope enrichment fractions of TCA cycle intermediates for parental and VEN-RE MOLM-13 cells. The glucose-derived ^13^C enrichment fraction (total of M + 2, +3, and +4) for fumarate (relative to succinate enrichment levels) shows slightly decreased levels in VEN-RE cells. Cit Citrate, Suc Succinate, Fum Fumarate, Mal Malate. **g** Results of Seahorse assays showing OCR in parental (dotted lines) and VEN-RE (solid lines) MOLM-13 cells treated with indicated inhibitors for 1 h. **h** Results of Seahorse assays showing OCR in VEN-RE MOLM-13 cells transfected with shCON (dotted lines) or shMCL-1 (solid lines) that were induced with or without doxycycline (100 ng/mL) for 6 h

Having demonstrated the role of MCL-1 in apoptotic priming of AML cells with acquired resistance to VEN, we then investigated additional alterations of the cellular functions governed by MCL-1. Considering the slower growth rate of VEN-RE cells and emerging reports on the involvement of BCL-2 family proteins in cell metabolism, we explored the total amounts and function of the mitochondria in VEN-RE cells. Quantification of the mitochondrial by real-time PCR demonstrated a reduced *ND-1* mitochondrial DNA copy number^21^ in VEN-RE cells (Fig. 3a). This was supported by transmission electron microscopy (TEM) analysis, which showed fewer mitochondria in these cells (Fig. 3b, c). Morphologically, the mitochondria in VEN-RE cells displayed altered crista structure (the inserts on Fig. 3c show zoomed images of the ultrastructure of random mitochondria), suggesting impaired mitochondrial function.

To validate this, we next examined mitochondrial respiration using Seahorse XF. While parental cells exhibited a dose-dependent increase in the maximum oxygen consumption rate (OCR) with mitochondrial uncoupling by carbonyl cyanide-4-(trifluoromethoxy) phenylhydrazone (FCCP), the VEN-RE cells had a lower maximum OCR and underwent minimal changes with increasing concentrations of FCCP, indicating lower spare oxidative phosphorylation capacity (Fig. 3d). On the contrary, there was no significant difference in the extracellular acidification rate (ECAR) between the parental and VEN-RE cells (Supplementary Fig. 3a). We next isolated mitochondria from paired parental and VEN-RE cells and tested their ETC complex activity. We found that VEN-RE MOLM-13 cells had reduced complex II function, but not complex I, III, IV (Fig. 3e and Supplementary Fig. 3b). These data indicate that the mitochondria in VEN-RE cells are compromised in both number and function.

To determine the effect of mitochondrial function on alteration of cell metabolism, we analyzed the metabolic differences between parental and VEN-RE MOLM-13 cells using a combination of LC-MS metabolomics and stable isotope metabolic tracing following enrichment with ^13^C_6_-glucose or ^13^C_5_,^15^N_2_-glutamine. The results illustrate an overall decrease in the pool levels of TCA cycle intermediates in VEN-RE cells (Fig. 3f), in line with the observed decreased mitochondria number and function. While the overall level of enrichment was lower in VEN-RE cells, the rate of both glucose- and glutamine-derived carbon enrichment (from the isotopically labeled substrates) remained largely unchanged between parental and VEN-RE cells. In agreement with the reduced activity of complex II (succinate dehydrogenase) observed in VEN-RE cells, the total glucose-derived carbon enrichment fraction of fumarate (as normalized to succinate enrichment) was approximately 7% lower in VEN-RE than in parental cells (*p* < 0.02).

Parental MOLM-13 cells treated with VEN exhibited the significant decrease in total levels of TCA cycle intermediates and closely related metabolites (e.g., 2HG and Asp), and these consistently remained unaltered in VEN-RE cells (Supplementary Fig. 3c, d). Labeling with ^13^C_6_-glucose or ^13^C_5_,^15^N_2_-glutamine overall resulted in minimal VEN treatment-induced changes in the rate of carbon enrichment. However, significant VEN-induced metabolic changes were observed for both the total level and the rates of carbon incorporation into several nucleotides, in particular monophosphate nucleotides. The rate of glucose and glutamine-derived carbon enrichment fraction dropped in parental VEN-treated cells pointing to a lower rate of de novo nucleotide biosynthesis (Supplementary Fig. 3c). Total metabolite levels, as well as rate of carbon incorporation remained unchanged in VEN-treated VEN-RE cells (Supplementary Fig. 3d).

Previous studies revealed that BCL-2 regulates oxidative phosphorylation in LSCs^14^, that the IMM form of MCL-1 regulates fatty acid oxidation^17^, and that the metabolic roles of these proteins are independent of their antiapoptotic functions. To understand whether MCL-1 regulates mitochondrial metabolism, we measured mitochondrial respiration upon S63845 or VEN treatment in parental and VEN-RE MOLM-13 cells; and upon inducible MCL-1 silencing with shMCL-1 or MCL-1 inhibition in VEN-RE MOLM-13 cells. Consistent with published reports, BCL-2 inhibition with VEN (10 nM, 1 h) inhibited OCR and ECAR in parental cells, but failed to affect mitochondrial activity in VEN-RE cells (Fig. 3g, Supplementary Fig. 3e). In turn, MCL-1 inhibition with S63845 (10 nM, 1 h) or inducible MCL-1 silencing (doxycycline 100 ng/ml, 6 h) reduced OCR in VEN-RE cells but not in parental cells (Fig. 3h, Supplementary Fig. 3f). These changes were evident prior to affecting mitochondria integrity in parental or in VEN-RE cells as measured by Cytochrome C flow cytometry (Supplementary Fig. 3g, h). These results indicate that in VEN-RE cells, MCL-1 maintains mitochondrial metabolism in addition to its antiapoptotic function, highlighting the shift of the mitochondrial functions of VEN-RE cells to MCL-1.

### MCL-1 upregulation is due to increased protein stability through RAS/MAPK activation

**Fig. 4:**
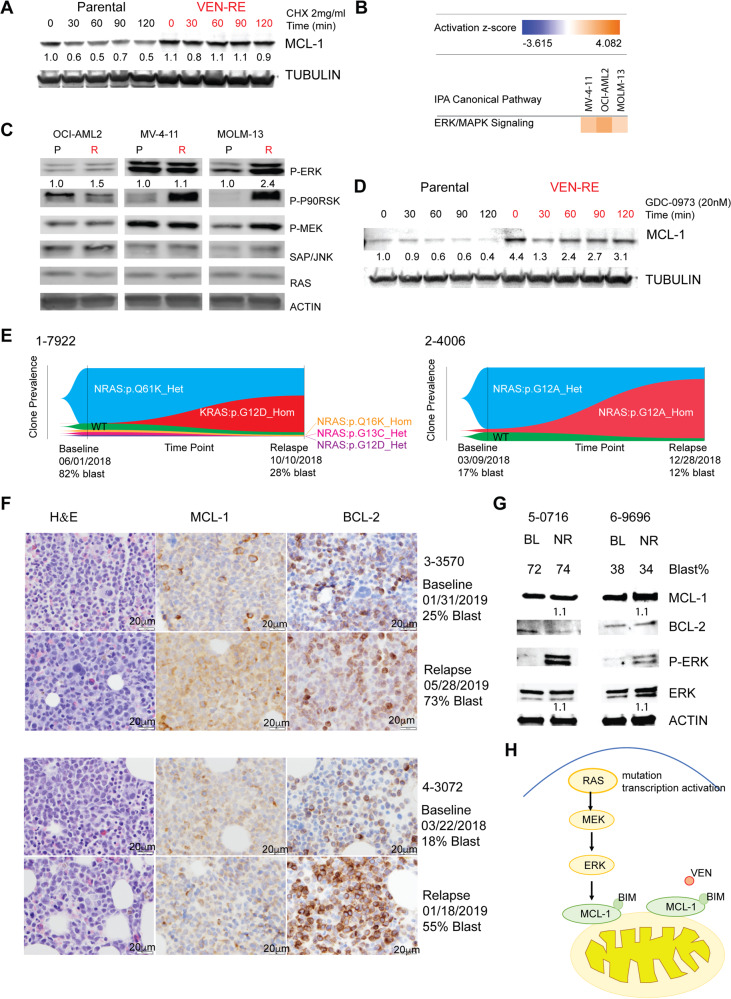
MAPK pathway activation in VEN-RE cells upregulated MCL-1. **a** Western blots showing MCL-1 protein levels in parental and VEN-RE MOLM-13 cells treated with 2 mg/mL cycloheximide (CHX) for 0–120 min. **b** Heatmap showing activation scores for ERK/MAPK signaling in VEN-RE cells based on pathway analysis of the RNA sequencing data. **c** Western blots showing protein levels of MAPK pathway components in parental (P) and VEN-RE (R) cells. **d** Western blots showing protein levels of MCL-1 in parental and VEN-RE MOLM-13 cells treated with 20 nM of the MEK inhibitor GDC-0973 (GDC) and 2 mg/mL CHX for 0–120 min. **e** Fishplots showing clone prevalence at two time points (baseline and relapse) in patient samples 1-7922 and 2-4006. **f** MCL-1 and BCL-2 expression by immunohistochemistry (IHC) in two paired samples from patients at the time of relapse on VEN clinical trial. Scale bar on the bottom right indicates 20 μm. **g** Protein expression in two AML patients primary refractory to VEN-based therapy pre- (BL) or post-treatment (no response, NR). **h** Mechanism of VEN resistance: RAS/MAPK/MCL-1 axis

We next addressed the underlying mechanisms of MCL-1 upregulation in VEN-RE cells. RNA sequencing found a 0.73 (log2) fold increase in *MCL-1* expression in VEN-RE OCI-AML2 and MOLM-13 cells, while MCL-1 protein increased in VEN-RE OCI-AML2 and MOLM-13 cells by 5.2- and 6.8-fold, respectively, suggesting alternative mechanisms. We first studied the contribution of post-translational modifications of MCL-1 to its upregulation. Treatment of parental cells with the protein synthesis inhibitor cycloheximide (2 mg/mL for up to 120 min) reduced MCL-1 protein levels in parental cells (MCL-1 estimated half-life 60 min) but not in VEN-RE cells, indicating increased protein stability in the VEN-RE cells (Fig. 4a). Notably, the MCL-1 level was gradually increased in VEN-RE cells at higher doses of VEN during selection (Supplementary Fig. 4a). These data strongly suggest that post-translational modification(s) increased MCL-1 stability and contributed to VEN resistance.

Pathway analysis of the RNA sequencing identified activation of the ERK/MAPK pathway in VEN-RE cells compared to parental cells (Fig. 4b). We validated activation of the MAPK pathway by immunoblotting, which demonstrated increased levels of p-P90RSK and p-ERK in VEN-RE cells (Fig. 4c). We and others have previously reported several mechanisms of MCL-1 stabilization by MAPK, through modulation of phosphorylation and protein degradation.^22^ We further showed that activation of MAPK rendered VEN-RE cells sensitive to several structurally diverse inhibitors of this pathway by small molecule inhibitor kinase screening (Supplementary Fig. 4b). We next tested the cellular and signaling responses of parental and VEN-RE OCI-AML2 cells to two MEK inhibitors, CI1040 and GDC-0973, and found that the VEN-RE cells maintained their sensitivity to MEK inhibition (Supplementary Fig. 4c). When cells were exposed to GDC-0973, the MCL-1 level rapidly (in 60 min) decreased in parental cells, while in VEN-RE cells, the MCL-1 level only minimally decreased and then returned to the baseline levels, suggesting activation of the pathway upstream of MEK (Fig. 4d).

A previous study found that BCL-2 could co-localize with NRAS, resulting in RAS activation,^23^ a known upstream activator of MAPK pathway. Of note, expansion of pre-existing RAS-mutant clones was reported recently in VEN-resistant AML pts, in association with monocytic phenotype.^24^ While we did not identify any mutations in RAS in our VEN-RE cells, they phenotypically exhibited RAS/MAPK activation, functionally resembling RAS-mutated genotype. Moreover, our RNAseq gene set enrichment analysis (GSEA) showed enrichment of genes that are targeted by KRAS, supporting activation of RAS signaling (Supplementary Fig. 4d, e).

Activation of the MAPK pathway through NRAS/KRAS mutations is a resistance mechanism commonly observed with targeted therapies, including FLT3, IDH and SYK inhibitors.^25–27^ Our recent report indicated similar enrichment of “signaling” mutations in patients progressing on VEN and low dose chemotherapy combination trials.^3^ To understand whether RAS-mutated clones exhibit fitness and resistance to VEN, we identified two patients who experienced disease relapse upon treatment with VEN/HMA combination and in whom targeted molecular sequencing detected a large RAS-mutant clone at the time of relapse. We utilized single-cell DNA sequencing by Tapestri technology to analyze clonal evolution in these patients. In patient #1-7922, a KRAS homozygous clone expanded from 0.02% prior to therapy (undetectable by conventional sequencing) to 47.6% at relapse after 4 months on therapy (Fig. 4f and Supplementary Table 3). Similarly, in patient #2-4006, an NRAS homozygous clone expanded from 4.6% at baseline to 80.7% at the time of relapse, after 9 months on therapy (Fig. 4f). We examined the MCL-1 and BCL-2 protein expression by IHC in two AML patients (#3-3570, #4-3072) harboring RAS mutation at the time of relapse on venetoclax combination trial, and found that MCL-1 expression increased in both patients at progression compared to baseline (Supplementary Table 4, Fig. 4f). We further detected increased pERK expression by immunoblotting in additional two patients (#5-0716, #6-9696) with RAS mutation refractory to venetoclax treatment after one cycle of treatment compared to baseline (Supplementary Table 4, Fig. 4g). We illustrate the identified mechanisms of RAS/MAPK/MCL-1 crosstalk in Fig. 4h, with genomic RAS mutation or epigenetic MAPK activation activating downstream MAPK signaling and stabilizing MCL-1, which in turn sequesters BIM from BCL-2, facilitates AML survival and maintains mitochondrial respiration, supporting cell metabolic fitness.

**Fig. 5:**
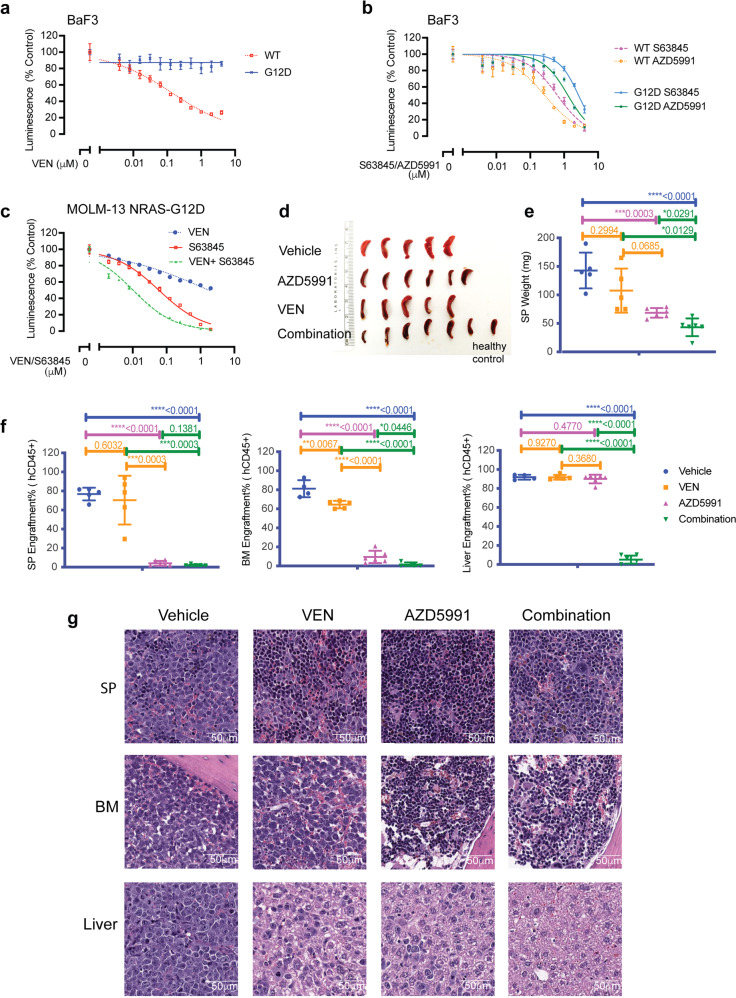
NRAS-G12D mutation confers resistance to VEN and sensitivity to MCL-1 inhibition. **a**, **b** The BaF3 cells expressing NRAS-WT (dotted lines) or NRAS-G12D (solid lines) were treated with VEN (**a**) or indicated MCL-1 inhibitors S63845 or AZD5991 (**b**) for 24 h, after which cell viability was determined by CellTiter-Glo assay. Luminescence reads were normalized to those of DMSO-treated control cells. Data represent mean ± SD from triplicate independent experiments. **c** MOLM-13 cells expressing NRAS-G12D were treated with indicated inhibitor for 24 h, then cell viability was determined by CellTiter-Glo assay. Luminescence reads were normalized to those of DMSO-treated control cells. Data are mean ± SD. **d** Spleen harvested from mice received vehicle, AZD5991, VEN, or the combination of AZD5991 and VEN. One spleen from a healthy mouse is shown for comparison. **e** Weight of spleens collected from each group. Data are mean ± SD. **f** Tumor burden shown by %hCD45 from spleens (SP), bone marrows (BM) and livers from each mouse in specified cohort. Data are mean ± SD. **g** H&E staining of SP, BM, and liver from one representative mouse from each group

To validate the effect of RAS/MAPK/MCL-1 pathway activation in mediating VEN resistance, we over-expressed NRAS-WT or NRAS-G12D^28^ in BaF3 cells (Supplementary Fig. 5a) and tested the sensitivity to BCL-2 inhibition with VEN, and to MCL-1 inhibition with S63845 and AZD5991. Compared to BaF3 cells with NARS-WT, the cells with NRAS-G12D were resistant to VEN, but maintained the sensitivity to S63845 and AZD5991 (Fig. 5a, b). Similarly, MOLM-13 NRAS-G12D cells were resistant to VEN, and inhibition of MCL-1 with S63845 restored cells sensitivity to VEN shown by the synergistic reduction in cell viability by the combination (Supplementary Fig. 5b, c, Fig. 5c).

We next examined the anti-leukemia efficacy of combination of VEN and AZD5991 in MOLM-13 NRAS-G12D cell line-derived (CLDX) xenograft model (Supplementary Fig. 5d). Both VEN, AZD5991 and their combination were well tolerated, as shown by stable body weight (Supplementary Fig. 5e). We previously reported that parental MOLM-13 CLDX was responsive to VEN.^19^ RAS-mutated AML in mice was refractory to VEN as demonstrated by negligible reduction in spleen tumor burden in spleen (SP) and rapid expansion of leukemia tumor burden in bone marrow (BM) and liver, consistent with the in vitro resistance to VEN (Fig. 5d, e, f, g, Supplementary Fig. 5f). In turn, AZD5991 and AZD5991/VEN combination therapy significantly decreased tumor burden in all compartments tested. Importantly, combinatorial therapy caused significant reduction in tumor burden compared with VEN or AZD5991 single agent cohorts, indicating that MCL-1 inhibition with AZD5991 restored VEN sensitivity in MOLM-13 NRAS-G12D CLDX (Fig. 5e, f, g, Supplementary Fig. 5f).

### Other contributors to VEN resistance identified by CRISPR screening

To identify additional potential mechanisms of VEN resistance, we performed an unbiased genome-wide CRISPR knockout screen (experimental design depicted in Supplementary Fig. 5e). We transduced the VEN-sensitive Cas9-expressing KO52 AML cell line with the Brunello library^29^ and treated the transduced cells with a high dose of VEN (10 μM) or DMSO as a control. Following a 2-week expansion of surviving cells, gRNAs were sequenced to identify enriched genes that confer resistance to VEN. Gene disruptions of *PMAIP1* (NOXA) or *BAX* were identified as the two most apparent hits that lead to VEN resistance (Supplementary Fig. 5h). *PMAIP1* and *BAX* are known proapoptotic regulators, and this finding agrees with recently published VEN resistance screens.^8,30^ Additionally, we confirmed our finding in two additional AML cell lines, MOLM-13 and OCI-AML2 (Supplementary Fig. 5i, j). MOLM-13 cells express FLT3-ITD fusion protein and OCI-AML2 cells have a mutation in *DNMT3A* gene, suggesting that loss of either *BAX* or *PMAIP1* may lead to VEN resistance irrespective on the cell’s genetic background. Notably, *PMAIP1* and *BAX* mutations were recently reported in CLL patients that relapsed on VEN monotherapy.^31^ Silencing of additional genes, including *OMA1*, *GLUL*, *KIAA0141*, and *CHST9* also lead to VEN resistance (Supplementary Fig. 5k). OMA1 is a metalloprotease that plays a role in the inner membrane of mitochondria. Upon induction of apoptosis induction and loss of mitochondrial membrane potential, OMA1 cleaves OPA1 protein leading to its inactivation and subsequent mitochondrial membrane reshaping and cytochrome *c* release.^32^ Interestingly, Chen et al.^8^ recently identified *OPA1* as a gene whose silencing leads to enhanced VEN resistance. These findings complement our analysis and indicate the importance of OMA1-OPA1 interaction in maintaining mitochondrial structure and apoptosis induction upon VEN treatment. Taken together, these findings support the notion that multiple alterations in apoptotic machinery, mitochondrial structural proteins, or cellular metabolism may represent diverse mechanisms of tumor escape from selective BCL-2 inhibition. Further studies are need to be performed to fully understand how these processes affect leukemia cell responses to VEN.

## Discussion

In this study, we investigated the mechanisms of acquired resistance to VEN in AML by generating multiple isogenic pairs of parental and VEN-RE cell lines through exposure to escalating doses of VEN. Our analysis revealed that cell-type-specific alterations in VEN-RE cells involve modulation of apoptosis-regulating genes at multiple levels: DNA methylation, transcription, and proteins expression. These findings highlight that multiple alterations in the apoptotic machinery may allow escape from targeted BCL-2 inhibition and support the need for combinatorial therapy already empirically adapted in AML clinical practice.

Our study highlighted the MCL-1 as a key mediator in VEN resistance. We found increased MCL-1 expression in VEN-RE cells by analyses of DNA methylation, mRNA expression (OCI-AML2 and MOLM-13), and protein level (all 3 VEN-RE cells). Furthermore, MCL-1 inhibition by inducible shRNA in VEN-RE cells (OCI-AML2 and MOLM-13) re-sensitized the cells to VEN. While the role of MCL-1 was reported previously,^5,33–35^ our analyses demonstrate the central role of RAS/MAPK pathway in the activation of MCL-1 in AML. We identified both, transcriptional enrichment of RAS-regulated genes in VEN-RE cells, the genomic expansion of *KRAS* and *NRAS* clones, and increased protein level of MCL-1 and p-ERK in patients progressing on VEN/HMA therapy. Further, we demonstrated that NRAS-G12D expressing BaF3 and MOLM-13 cells became resistant to VEN in vitro and in vivo, and that the inhibition of MCL-1 restored MOLM-13 NRAS-G12D cells sensitivity to VEN. Additional components of MAPK signaling, such as mutations in negative regulator phosphatase *PTPN11*, were reported by us in AML patients refractory to or relapsing on single agent VEN therapy,^36^ similar to expanding clones containing mutations in FLT3-ITD, and upstream regulator of MAPK activity, in both single agent and in HMA combination trials.^36,37^ These findings indicate the utility of co-targeting MAPK and BCL-2, demonstrated by our group previously^38^ and confirmed here in VEN-RE cells by kinase screening tool, the finding that lends support for testing the combinations in clinical trials (NCT02670044, NCT04487106).

Our study further identified multiple additional mechanisms of VEN resistance in the pathways controlling cell death and mitochondrial biology, which were commonly cell-type-specific. In VEN-RE MOLM-13 cells alterations in TNF/TRAIL pathway components, such as increased *LMNA* and *APAF1* and decreased *TNFRSF8*, *TNFSF10*, *FHIT*, and *RIPK1* mRNA expression, were prominent. The modulation of TRAIL pathway was reported to mediate resistance to BCL-2 inhibition in solid tumors and CLL,^35,39^ which was also identified by CRISPR screening in our study.

We have further found the mitochondrial protease OMA1, which interacts with OPA1 and maintains mitochondrial architecture as well as lipid metabolism,^40^ as a top resistance-related factor by CRISPR screening. This agrees with a recent report on VEN resistance in AML,^8^ which discovered the mitochondrial chaperone CLPB that interacts with OPA1, maintaining physiological mitochondrial structure. Consistent with published reports, VEN reduced OCR in parental but not in resistant cells. Utilizing metabolomic tracing experiments, we demonstrated VEN-triggered reduction of total pools of TCA cycle metabolites, 2-HG and Aspartate, and reduced incorporation of glucose and glutamine-derived carbon into nucleotides in VEN-sensitive cells, supporting an important role of BCL-2 in mitochondrial metabolism in addition to its conventional pro-survival function. Our study, in contrast to recent report in VEN-RE lymphoma,^35^ found reduced basal and maximal OCR, decreased mitochondria number with dysmorphic cristae and reduction of TCA cycle intermediates in VEN-RE AML cells overexpressing MCL-1. Consistently, silencing of MCL-1 by shRNA in VEN-RE cells reduced OCR, suggesting that in AML cells MCL-1 partially compensates for BCL-2’s role in cellular respiration, yet maintaining lower oxidative capacity. Analysis of the components of ETC chain and metabolomics analysis demonstrated the reduced function of complex II, recently shown to be directly targeted by VEN.^41^ Notably, it was reported that the OGT-deficient cells had impaired mitochondrial morphology with decreased spare respiratory capacity and significant loss of mitochondria content,^42^ and our results identified a missense mutation of *OGT* in VEN-RE MOLM-13 and OCI-AML2 cells, a finding that requires further exploration. Combined, these results support the notion of disturbed mitochondrial fitness in VEN-RE AML cells, which may represent “Achilles heel” amenable to therapeutic interventions. These findings further support the importance of non-apoptotic functions of BCL-2 and MCL-1, prompting detailed evaluation of the functional contribution of these to sensitivity or resistance in patients.

We reported substantial genome-wide changes in DNA methylation in VEN-RE cells, quite an unexpected finding. Although there are some reports that the DNA methylation signature regulates apoptosis in cancer cells,^43^ no studies have shown a link between apoptosis-related genes such as BCL-2 or MCL-1, and DNA methylation. HMA treatment of the aberrantly-methylated VEN-RE cells restored moderate sensitivity to VEN, suggesting that AML patients may benefit from the VEN and HMA combination treatment, which has indeed been observed in clinical trials.^44^ While our study highlighted the epigenetic function of HMA in mediating the sensitivity to VEN. A recent study reported a non-epigenetic role of HMA in sensitizing the cells to VEN through induction of NOXA.^45^

There are few notable limitations of our study. While we established three AML cell line models with different genomic backgrounds and increased the sample size and power of our analysis by selecting single-cell clones, only a few targets were shared across the tested cell lines. We focused on OCI-AML2 and MOLM-13 cell lines, which represent different genomic backgrounds (*NPM1/ FLT3* WT in OCI-AML2 vs. *FLT3-ITD* mutant in MOLM-13), yet these two cell lines did show certain similarities. Future work focusing on VEN resistance in different models may help identify context-specific resistance mechanisms.

In conclusion, our study identified complex mechanisms underlying acquired resistance to VEN in AML. Our data support the model that RAS/MAPK activation upregulates MCL-1, which sequesters BIM from BCL-2 and becomes the dominant antiapoptotic protein maintaining mitochondrial metabolism and survival under BCL-2 inhibition. By several orthogonal methods, our results point out to mitochondrial fitness as a major downstream determinant of sensitivity or resistance to BCL-2 blockade. Combination therapies targeting RAS/MCL-1 and BCL-2, or direct co-targeting of mitochondria and BCL-2, may represent future directions in AML therapy.

## Materials and methods

### Drugs

Venetoclax (VEN), A-1155463, and A-1210477 were provided by AbbVie. GDC-0973 (cobimetinib), CI1040, ABT-737, S63845, and S55746 were purchased from Selleckchem. Cycloheximide and doxycycline were purchased from Sigma-Aldrich.

### Cell culture and generation of VEN-RE cell lines

OCI-AML2 and MOLM-13 AML cell lines were purchased from Deutsche Sammlund von Mikroorganismen und Zellkulturen. MV-4-11 cell lines were purchased from ATCC. Ko52 cell line was purchased from AcceGen. All cells were authenticated by short tandem repeat DNA fingerprinting in September 2016 by the Cytogenetics and Cell Authentication Core Facility at The University of Texas MD Anderson Cancer Center. All cell lines were cultured in suspension in RPMI-1640 medium (Sigma) supplemented with 10% fetal bovine serum (Sigma), L-glutamine, and penicillin/streptomycin (Invitrogen). VEN resistance was induced by continuously exposing the parental cells to increasing concentrations of VEN, starting at 10 nM and increasing to 1 μM. The medium with VEN was changed every 2 days. After cells achieved stable viability (above 90%) at the current VEN dose, the dose was increased until it reached 1 μM. For single-clone selection, VEN-RE cells were seeded into 96-well plates at one cell/well by limited dilution assay. Three days later, wells with single-cell clones were identified under a microscope. After 7 to 10 days, the single clones were transferred to 48-well plates, and then to larger flasks for expansion until a sufficient number of cells for use in further experiments was reached. The single-cell clones were maintained in 1 μM VEN during selection.

### CellTiter-Glo assay

Cells were treated with inhibitors at the indicated concentrations and time points in 96-well plates. The cells were then mixed with CellTiter-Glo reagent (Promega) according to the manufacturer’s instructions. The reads from the treated wells were normalized to those of control wells. Inhibition curves were generated by using GraphPad Prism 7.0.

### Stable isotope tracing metabolomic analysis

Cells were seeded under three different labeling conditions: unlabeled glucose and glutamine, ^13^C_6_-glucose and unlabeled glutamine, or unlabeled glucose and ^13^C_5_,^15^N_2_-glutamine.^46^ Following a 6 h treatment, cells were aspirated, washed with phosphate buffered solution, harvested by centrifugation, and snap frozen in liquid nitrogen. Metabolite extraction was performed using a modified Bligh-Dyer extraction as previously described.^46^ The polar fraction was transferred to LC-MS vials for immediate analysis. Metabolite extracts were spiked with a mixture of deuterated internal standards (IS) to monitor retention time, ionization efficiency, and instrument stability. Chromatographic separation was achieved on an Accela 1250 ultrahigh pressure liquid chromatography (UPLC) system as previously described.^46,47^ The eluent was coupled to a Q Exactive Hybrid Quadrupole Orbitrap mass spectrometer with an electrospray ionization (ESI) source simultaneously operating in fast negative/positive ion switching mode (Thermo Scientific, Bremen, Germany) with the acquisition settings previously reported.^46,47^ Raw files were processed using SIEVE 2.2.0 SP2 (Thermo Scientific, San Jose, CA, USA) and in-house scripts operating in the MATLAB programming environment. Metabolite identification was determined by matching accurate masses and retention times to an in-house library of authentic standards that includes the IROA 300, MS Metabolite Library of Standards (IROA Technologies, Bolton, MA). Peaks were included in analysis if the coefficient of variance (CV) was <0.25 and >0 in the QC replicates. Probabilistic quotient normalization (PQN) was performed prior to statistical and metabolic analyses.^48^

### Reverse phase protein array (RPPA), western blotting and co-IP

RPPA was performed at MDACC Core Facility using all antibodies available in the core. RPPA methodology was described previously (42). For multiple markers, Western blotting was conducted according to previously published procedures.^38^ The same amounts of cell lysates were loaded in each membrane for each target. Actin was used as a loading control. The antibodies used for Western blotting were BCL-2 (Agilent DAKO), MCL-1 (Santa Cruz Biotechnology), BCL-XL, BAX, BAK, PUMA (Cell Signaling Technology), BCL-2 A1, NOXA, BIM (Abcam), actin, and tubulin (Sigma). The antibodies used for co-IP were BIM, mouse immunoglobulin G (Santa Cruz Biotechnology), and MCL-1 (Millipore Sigma). Co-IP experiments were performed using a Dynabeads Protein A Immunoprecipitation Kit (ThermoFisher) following the manufacturer’s instructions. In brief, cells were washed with cold phosphate buffered saline and lysed with cell lysis buffer (Cell Signaling Technology), then the cleared lysates were incubated with antibody-conjugated Dynabeads. The bead-protein complexes were then isolated using a magnet and washed. The proteins were later eluted from the beads and used for subsequent experiments.

### BH3 profiling

This assay was conducted as previously reported.^18^ In brief, cells were pelleted at 400 *g* and resuspended in DTEB medium. Cells were then permeabilized with digitonin and exposed to BH3 peptides (BIM, PUMA, FS-1, NOXAA, MS1, BAD, HRK, synthesized by New England Peptide). The mitochondrial transmembrane potential loss was monitored using the ratiometric dye JC-1 (Invitrogen).

### Inducible MCL-1 knockdown

An shRNA targeting MCL-1 (shMCL-1: sense: ccggtgtgaattcatgggctcatcttcaagagagatgagcccatgaattcacttttttgg; antisense: aattccaaaaaagtgaattcatgggctcatctctcttgaagatgagcccatgaattcaca) was used to knockdown MCL-1 expression. A hairpin targeting green fluorescent protein in pLKO.1 was used as control (Addgene). The doxycycline-inducible knockdown system was previously reported.^49^ In brief, a lentivirus was prepared by co-transfection of HEK293T cells with an equal-molar mix of vector and packaging plasmids (psPAX2 and pMD2.G, Addgene) using JetPrime transfection reagent according to the manufacturer’s instructions (Polyplus). Fresh lentiviral supernatants were collected and aliquoted as stock in a freezer at −80 °C. The cells were spinoculated with virus and polybrene and then selected by 0.5 μg/mL puromycin.

### Mitochondrial DNA copy number analysis

The relative mitochondrial DNA copy number was measured by using a quantitative reverse-transcription PCR-based method.^21^ Two primers were used: the *ND-1* primer pair ND1-F, 5′-CCCTAAAACCCGCCACATCT-3′, ND1-R, 5′-GAGCGATGGTGAGAGCTAAGGT-3′; and the *HGB* primer pair HGB-F, 5′-GTGCACCTGACTCCTGAGGAGA-3′, HGB-R, 5′-CCTTGATACCAACCTGCCCAG-3′. The ratio of the number of mt*ND-1* genes to the number of nuclear *HGB* genes in VEN-RE cells was normalized to that of the parental cells.

### Transmission electron microscopy (TEM)

TEM was performed at the High Resolution Electron Microscopy Facility at MD Anderson Cancer Center. Samples were fixed with a solution containing 3% glutaraldehyde plus 2% paraformaldehyde in 0.1 M cacodylate buffer, pH 7.3, then washed in 0.1 M sodium cacodylate buffer, treated with 0.1% Millipore-filtered cacodylate-buffered tannic acid, postfixed with 1% buffered osmium, and stained *en bloc* with 1% Millipore-filtered uranyl acetate. The samples were dehydrated in increasing concentrations of ethanol, infiltrated, and embedded in LX-112 medium. The samples were polymerized in an oven at 60 °C for approximately 3 days. Ultrathin sections were cut using a Leica Ultracut microtome, stained with uranyl acetate and lead citrate in a Leica EM Stainer, and examined using a JEM 1010 transmission electron microscope (JEOL USA, Inc.) at an accelerating voltage of 80 kV. Digital images were obtained using the AMT Imaging System (Advanced Microscopy Techniques Corp.).

### Seahorse mitochondrial stress assays

Cells were seeded with Seahorse medium (Agilent, Seahorse XF medium with 2 mM glutamate, 10 mM glucose, and 2 mM pyruvate) in 96-well Seahorse plates. Plates were centrifuged at 1200 *g* to settle cells to the bottom of the wells. Mitochondrial stress tests were then performed with 1.5 μM oligomycin, 0.25–2.5 μM FCCP, and 0.5 μM rotenone and antimycin on a Seahorse XFe 96 Analyzer (Agilent). Reads were normalized to the viable cell count. A detailed protocol is provided at Agilent’s website: https://www.agilent.com/cs/library/usermanuals/public/insert-xf-pmp-reagent-web.pdf. Data were normalized to per one million cells.

### Single-cell DNA sequencing

Patient peripheral blood or bone marrow samples were collected under an IRB-approved protocol and with written consent. The protocol adhered to the Declaration of Helsinki. The assay was previously reported.^50^ In brief, the single-cell suspensions were prepared, cells were first encapsulated into droplets, lysed, protease-treated, and secondly, after heat-inactivating the protease, combined with PCR reagents and Barcoding Beads using Mission Bio’s microfluidics devices and the Tapestri platform. Next, after highly parallel multiplex targeted PCR amplification, single-cell emulsions were then broken, amplified DNA collected, and purified with Ampure XP reagent (Beckman Coulter). After adding Ilumina indices and P5/P7 adapters via 10-cycle PCR, the DNA products were purified again (Ampure XP) to obtain full-length amplicons. Library size distributions and concentrations were analyzed on a DNA 1000 assay chip using Bioanalyzer (Agilent) and sequenced on an Illumina MiSeq sequencer. The data were analyzed with Tapestri Insights software version 2.1 (Mission Bio) using default settings.

### Generation of *NRAS-G12D* expressing cells

GFP + BaF3 or MOLM-13 cells were sorted by flow cytometry 3 days after retroviral infection with *NRAS-G12D* vector^28^ (same virus packing as in inducible shMCL-1 knockdown section). *NRAS-G12D* expression were induced with doxycycline (1ug/ml) for 72 h prior experiment.

### Animal studies

All mouse experiments were approved by the Institutional Animal Care and Use Committee at MD Anderson Cancer Center. 8-week-old NOD/SCID gamma (NSG) mice (Jackson Laboratory) were injected with 0.5 × 10^6^ MOLM-13 NRAS-G12D cells via the tail vein. Mice received 2 mg/ml doxycycline in 1% sucrose water for NRAS-G12D expression induction throughout the experiment. Mice were randomized to receive vehicle, venetoclax (100 mg/kg, po., qd.), AZD-5991 (100 mg/kg, iv., qw.), or combination of the two for 2 weeks after confirmed engraftment by bone marrow flow cytometry 3 days after injection. Mice organs (liver, spleen, and femur) were collected after euthanization. Tumor burden was quantified by hCD45+ flow cytometry.

### Statistical analysis

Statistical analyses indicated in each figure were performed using GraphPad Prism 7.0. Results are shown as mean ± SD of representative experiments from at least three independent experiments unless otherwise indicated. Differences with *P* ≤ 0.05 were considered statistically significant. *P < 0.05, **P < 0.005, ***P < 0.0005, ****P < 0.0001.

## Supplementary information

Supplementary Material

## Data Availability

The authors declare that the data supporting the findings of this study are available within the paper and its Supplementary materials.
