# Correction: Activation of RAS/MAPK pathway confers MCL-1 mediated acquired resistance to BCL-2 inhibitor venetoclax in acute myeloid leukemia

**DOI:** 10.1038/s41392-022-00958-4

**Published:** 2022-04-01

**Authors:** Qi Zhang, Bridget Riley-Gillis, Lina Han, Yannan Jia, Alessia Lodi, Haijiao Zhang, Saravanan Ganesan, Rongqing Pan, Sergej N. Konoplev, Shannon R. Sweeney, Jeremy A. Ryan, Yulia Jitkova, Kenneth Dunner, Shaun E. Grosskurth, Priyanka Vijay, Sujana Ghosh, Charles Lu, Wencai Ma, Stephen Kurtz, Vivian R. Ruvolo, Helen Ma, Connie C. Weng, Cassandra L. Ramage, Natalia Baran, Ce Shi, Tianyu Cai, Richard Eric Davis, Venkata L. Battula, Yingchang Mi, Jing Wang, Courtney D. DiNardo, Michael Andreeff, Jeffery W. Tyner, Aaron Schimmer, Anthony Letai, Rose Ann Padua, Carlos E. Bueso-Ramos, Stefano Tiziani, Joel Leverson, Relja Popovic, Marina Konopleva

**Affiliations:** 1 Department of Leukemia, The University of Texas MD Anderson Cancer Center, Houston, TX USA; 2 AbbVie Inc., North Chicago, IL USA; 3 Institute of Hematology, Chinese Academy of Medical Sciences & Peking Union Medical College, Tianjin, China; 4 Department of Nutritional Sciences, Department of Pediatrics, Department of Oncology, Dell Medical School, The University of Texas at Austin, Austin, TX 78712 USA; 5 Department of Cell, Developmental & Cancer Biology, Division of Hematology & Medical Oncology, Knight Cancer Institute, Oregon Health & Science University, Portland, OR USA; 6 Université de Paris, Institut de la Recherche Saint-Louis (IRSL), Inserm Unit 1131, Paris, France; 7 Dana-Farber Cancer Institute, Boston, MA USA; 8 Department of Hematopathology, The University of Texas MD Anderson Cancer Center, Houston, TX USA; 9 Princess Margaret Cancer Center, Toronto, ON Canada; 10 High Resolution Electron Microscopy Facility, The University of Texas MD Anderson Cancer Center, Houston, TX USA; 11 Department of Bioinformatics & Computational Biology, The University of Texas MD Anderson Cancer Center, Houston, TX USA; 12 Department of Hematology, The First Hospital Affiliated Harbin Medical University, Harbin, China; 13 Department of Lymphoma & Myeloma Research, The University of Texas MD Anderson Cancer Center, Houston, TX USA

**Keywords:** Haematological cancer, Translational research

Correction to: *Signal Transduction and Targeted Therapy* (2022) **7**:1–13, 10.1038/s41392-021-00870-3

In the original version of this article, given name of 4th author Yannan Jia was incorrectly published as Yanan Jia.

The original article has been corrected.

