# Supplementary material for: Activation of RAS/MAPK pathway confers MCL-1 mediated acquired resistance to BCL-2 inhibitor venetoclax in acute myeloid leukemia
Source: Signal Transduct Target Ther. 2022 Feb 21;7:51. doi: 10.1038/s41392-021-00870-3 (PMC8858957; doi:10.1038/s41392-021-00870-3)
Supplement: Supplementary file 1 — Supplementary Material [file 41392_2021_870_MOESM1_ESM.pdf]

# Supplementary Materials for

**Activation of RAS/MAPK pathway confers MCL-1 mediated acquired resistance to BCL-2 inhibitor venetoclax in acute myeloid leukemia**

Qi Zhang[1], Bridget Riley-Gillis[2], Lina Han[1], Yanan Jia[1,3], Alessia Lodi[4], Haijiao Zhang[5], Saravanan Ganesan[6], Rongqing Pan[7], Sergej N Konoplev[8], Shannon R.Sweeney[4], Jeremy A. Ryan[7], Yulia Jitkova[9], Kenneth Dunner Jr.[10], Shaun E. Grosskurth[2], Priyanka Vijay[2], Sujana Ghosh[2], Charles Lu[2], Wencai Ma[11], Stephen Kurtz[5], Vivian R. Ruvolo[1], Helen Ma[1], Connie C. Weng[1], Cassandra L. Ramage[1], Natalia Baran[1], Ce Shi[1,12], Tianyu Cai[1], Richard Eric Davis[13], Venkata L. Battula[1], Yingchang Mi[3], Jing Wang[11], Courtney D DiNardo[1], Michael Andreeff[1], Jeffery W. Tyner[5], Aaron Schimmer[8], Anthony Letai[7], Rose Ann Padua[6] , Carlos E. Bueso-Ramos[8], Stefano Tiziani[4], Joel Leverson[2], Relja Popovic[2], Marina Konopleva[1#]

[1] Department of Leukemia, The University of Texas MD Anderson Cancer Center, Houston, Texas, USA

[2] AbbVie Inc., North Chicago, Illinois, USA

[3] Institute of Hematology, Chinese Academy of Medical Sciences & Peking Union Medical College, Tianjin, China

[4] Department of Nutritional Sciences, Department of Pediatrics, Department of Oncology, Dell Medical School, The University of Texas at Austin, Austin, TX, 78712, USA

[5] Department of Cell, Developmental & Cancer Biology, Division of Hematology & Medical Oncology, Knight Cancer Institute, Oregon Health & Science University, Portland, Oregon, USA

[6] Université de Paris, Institut de la Recherche Saint-Louis (IRSL), Inserm Unit 1131, Paris, France

[7] Dana-Farber Cancer Institute, Boston, Massachusetts, USA

[8] Department of Hematopathology, The University of Texas MD Anderson Cancer Center, Houston, Texas, USA

[9] Princess Margaret Cancer Center, Toronto, Ontario, Canada

[10] High Resolution Electron Microscopy Facility, The University of Texas MD Anderson Cancer Center, Houston, Texas, USA

[11] Department of Bioinformatics & Computational Biology, The University of Texas MD Anderson Cancer Center, Houston, Texas, USA

[12] Department of Hematology, The First Hospital Affiliated Harbin Medical University, Harbin, China

[13] Department of Lymphoma & Myeloma Research, The University of Texas MD Anderson Cancer Center, Houston, Texas, USA

Correspondence to: mkonople@mdanderson.org

**This PDF file includes:**

Materials and Methods
Figures. S1 to S5
Tables S1 to S4

## Materials and Methods

**Whole-genome sequencing**: DNA samples were prepared in accordance with the manufacturer's instructions for the KAPA Hyper Prep kit. Briefly, 100ng of DNA was sheared using a Covaris M220 sonicator (adaptive focused acoustics). DNA fragments were end-repaired, adenylated, ligated with KAPA dual indexing adapters, and amplified by 11 cycles of PCR with the KAPA Hyper Prep kit. Final libraries were evaluated using Qubit (ThermoFisher) and Agilent Tape Station and were sequenced on an Illumina HiSeq 3000 sequencer using 2x150bp read length.

Whole genome sequencing reads were processed through the Sentieon TNhaplotyper pipeline (Sentieon Inc.) for alignment and somatic variant calling (Parental sample designated as "normal", resistant samples designated as "tumor"). Quality of sequencing data was assessed using Picard (http://broadinstitute.github.io/picard) and MultiQC [1]. SNV and indel variant calls were imported into the VarSeq software (Golden Helix) for filtering and annotation.

**RNA sequencing**: Each cell line was sampled in triplicate and RNA library preparation from total RNA was conducted following the manufacturer's protocol for the Kapa RNA HyperPrep Kit with RiboErase (HMR). Briefly, 400ng of total RNA was depleted of rRNA by hybridization of complementary DNA oligos, followed by treatment with RNase H and DNAase to remove hybridized rRNA. The remaining RNA was then fragmented by divalent cations under elevated temperature. The fragmented RNA underwent first strand synthesis using reverse transcriptase and random primers. Combined second strand synthesis and A-tailing incorporate dUTP into the second cDNA strand for stranded RNA sequencing and added dAMP to the 3' ends. The cDNA fragments were then ligated to sequencing adaptors (IDT xGEN Dual Index UMI adapters) and

the library was enriched using 9 cycles of PCR. Final libraries were assessed using the Agilent Tapestation and Qubit (ThermoFisher) assay methods then sequenced on an Illumina HiSeq 4000 sequencer using 2x75bp read length.

RNA sequencing reads were mapped to the human reference genome (GRCh38) using STAR aligner and genes were quantified using featureCounts for all genes annotated in Gencode v28 [2,3]. Quality of sequencing data was assessed using Picard (http://broadinstitute.github.io/picard) and MultiQC [1]. Genes with counts per million less than 1 in two-thirds of samples or more were considered too lowly expressed and excluded. Differential gene expression (DGE) analysis comparing parental vs. resistant was then performed using linear modeling in limma with TMM normalization and voom transformation [4] DGE results were plotted using glimma [5].

**DNA methylation analysis:** Illumina EPIC methylation array analyses were conducted with R statistical software (Version > = 3.5) (R Core Team, 2017). Bead count and detection p-value filtering were performed using the pfilter function from the watermelon R package with default settings [6] The getQC and plotQC functions from the minfi package [7] were used with default settings to check that all samples had sufficiently high median intensities. EPIC arrays were dasen normalized which performs background adjustment, between-array normalization for the Type I and Type II probes separately, and no dye bias correction. Beta values were calculated using functions in wateRmelon. Differentially methylated positions were identified by linear modeling and performing contrasts using the limma R package [8].

**Mitochondrial enzymatic assays**: This assay was performed as previously reported [9]. Complex I activity was detected by monitoring rotenone-sensitive 2,6-dichloroindophenol reduction by electrons accepted from decylubiquinol reduced after oxidation of NADH by complex I [10]. Complex II activity was measured by monitoring malonate-sensitive reduction of 2,6-dichloroindophenol when coupled to complex II-catalyzed reduction of decylubiquinol [11]. Complex III activity was determined with a modified method described previously [12,13]. The oxidation of decylubiquinols by complex III was determined using 6 cytochrome c as the electron acceptor. Reduced decylubiquinol and 5 to 10 μg of mitochondrial protein were added to the assay medium (35 mM $KH_2PO_4$, pH 8.0, 5 mM $MgCl_2$, 2 mM KCN, 50 μM rotenone, 0.1 mg/mL bovine serum albumin). The reaction was started by adding oxidized cytochrome c. Complex III specific activity was measured by determining the increase in absorbance due to the reduction of cytochrome c at 550 nm, with 580 nm as the reference wavelength. Complex IV activity was measured by KCN-sensitive oxidation of ferrocytochrome c [14]. Ferrocytochrome c was prepared by reducing cytochrome c with sodium ascorbate followed by dialysis for 24 h [15]. The method of assessing citrate synthase activity was based on the chemical coupling of CoASH, released from acetyl-CoA during the enzymatic synthesis of citrate to DTNB (Ellman's reagent, 5,5′-dithiobis (2-nitrobenzoic acid)), and the release of the absorbing mercaptide ion was monitored at 412 nm. The enzyme activity of Complexes I, II, and IV was normalized to citrate synthase activity, and notated as nmol/min/mg/citrate synthase activity.

**Ultrahigh performance liquid chromatography-mass spectrometry (UPLCMS) analysis**: Metabolite extraction was performed using a modified Bligh-Dyer extraction[16]. Briefly, cell pellets were extracted with 1:1 water:methanol with 10 mM ammonium bicarbonate and equal

parts chloroform. The polar fraction was transferred to LC-MS vials for immediate analysis. Metabolite extracts were spiked with a mixture of deuterated internal standards (IS) to monitor retention time, ionization efficiency, and instrument stability. Chromatographic separation was achieved on an Accela 1250 UPLC system equipped with a quaternary pump, vacuum degasser, and an open autosampler with temperature controller (6°C, Fisher Scientific, San Jose, CA, USA) coupled to a UPLC column. A Synergi 4 µm Hydro-RP 80 Å, 150 x 2 mm HPLC column (Phenomenex, Torrance, CA, USA) was used for metabolite separation. Mobile phase A was water with 0.2% formic acid and mobile phase B was methanol. A gradient separation was performed holding 1% B for 2 minutes, then linear 30-80% B in 8 minutes, washing with 98% B for 5 minutes, and column equilibration with 1% B for 15 minutes. The total run time was 30 minutes with a flow rate of 250 µL/min and an injection volume of 5 µL. The eluent was coupled to a Q Exactive Hybrid Quadrupole Orbitrap mass spectrometer with an electrospray ionization (ESI) source simultaneously operating in fast negative/positive ion switching mode (Thermo Scientific, Bremen, Germany). The following acquisition settings were used for data collection in full MS mode: spray voltage, 3.5 kV; capillary temperature, 320°C; sheath gas, 45 (arbitrary units); auxiliary gas, 10 (arbitrary units); m/z range, 50-750 ; data acquisition, centroid mode; microscans, 10; AGC target, 1e6; maximum injection time, 200 ms; mass resolution, 70,000 FWHM at m/z 200. Accuracy of MS analysis was ensured by calibrating the detector prior to analysis. The UPLC-MS analytical platform was controlled by a computer operating the Xcalibur v. 2.2 SP1.48 software package (Thermo Scientific, San Jose, CA, USA). Raw files were processed using SIEVE 2.2.0 SP2 (Thermo Scientific, San Jose, CA, USA) and an in-house scripts operating in the MATLAB programming environment. Metabolite identification was determined by matching accurate masses and retention times to library of standards. Peaks were

included in analysis if the coefficient of variance (CV) was >0 and < 0.25 in the QC replicates. Probabilistic quotient normalization (PQN) was performed prior to statistical analysis[17].

**Genome-wide ABT-199 resistance CRISPR knock-out screen:** KO52-Cas9 stable cells were generated by infecting the parental cells with lentivirus expressing Cas9-2A-mCherry and enriched by sorting for the high mCherry (high Cas9) expressers. The cells were tested for Cas9 activity by infecting them with the EGFP-2A-Puromycin cassette which also expressed the sgRNA targeting EGFP. The Brunello library containing sgRNAs targeting 19,114 genes (77,441 sgRNAs, average 4 sgRNAs per gene and 1000 non-targeting control sgRNAs) was packaged into lentivirus in HEK 293T cells using the VSVG and PAX2 plasmids. For each replicate, 140 million KO52-Cas9 cells were spin-fected in 12- well plates at 500 g at $30^{0}$C for 2 h in the presence of 6ug/ml polybrene (Sigma Aldrich) and Lentiblast (A:B 1:10) (OZBiosciences) with a multiplicity of infection (MOI) of 0.3 to ensure each cell received 1 sgRNA containing virus. The plates were then returned to the $37^{0}$C incubator with 5% CO2. Cells were incubated overnight, followed by pooling of each of the three biological replicates and then re-seeded into 175 ml non-tissue culture treated flasks (Nunc, Thermo Fisher). 48 h later (72 hours post infection) cells were selected with puromycin at 2ug/ml. 4 days later dead cells were removed using the Dead Cell removal kit (Miltenyi Biotec), infection efficiency was calculated using in-line assay and cells were re-seeded maintaining 40 million cells per replicate (to ensure ~ 500 fold library coverage). After two weeks of puro selection, each replicate was split into two treatment arms (40 million cells), 10uM ABT-199 and DMSO. 24 h post treatment dead cells were removed (> 88% of cells) and the live cells were expanded for an additional two weeks and collected. Genomic DNA was extracted using the QIAamp DNA Blood Maxi and Midi Kits (Qiagen)

according to manufacturer's instruction. The sgRNA sequences were amplified using the primers harboring sequencing adaptors and barcodes. Multiple PCR's covering all the genomic DNA were performed according to protocols from the Broad Institute. Each PCR reaction was performed with 10ug of genomic DNA using ExTaq DNA Polymerase (Clontech). Amplified sgRNAs were purified with AMPure XP-beads (Beckman Coulter) according to manufacturer's instructions. Samples were sequenced on the Illumina NextSeq 500 platform (1X 75bp). Analysis of the CRISPR screen was performed using internally developed pooled CRISPR workflow. Briefly, quantification of the sgRNA for each sample was carried out using internally developed Perl script. Normalization all samples was carried out using TMM algorithm [18]. Differential count comparison at the sgRNA level was done using Limma Bioconductor package [8]. Statistical tests at the gene level was done using internally implemented version of the Robust Rank Aggregation (RRA) [19].

**Small Molecule Inhibitor Screen**: Small-molecule inhibitors, purchased from LC Laboratories (Woburn, MA) and Selleck Chemicals (Houston, TX), were reconstituted in DMSO and stored at −80 °C. Inhibitors were distributed into 384-well plates prepared with a single agent per well in a seven-point concentration series ranging from 10 μM to 0.0137 μM for each drug. The final concentration of DMSO was ≤0.1% in all wells. Cells were seeded into 384-well assay plates at 1250 cells per well in RPMI 1640 media supplemented with FBS (20%), L-glutamine, and penicillin/streptomycin. After 3 d of culture at 37 °C in 5% $CO_2$, MTS reagent (CellTiter96, Promega, Madison, WI) was added, optical density was measured at 490 nm, and raw absorbance values were adjusted to a reference blank value and then used to determine cell viability (normalized to untreated control wells)[20].

## References


1 Ewels, P., Magnusson, M., Lundin, S. & Kaller, M. MultiQC: summarize analysis results for multiple tools and samples in a single report. *Bioinformatics* **32**, 3047-3048, doi:10.1093/bioinformatics/btw354 (2016).

2 Dobin, A. *et al.* STAR: ultrafast universal RNA-seq aligner. *Bioinformatics* **29**, 15-21, doi:10.1093/bioinformatics/bts635 (2013).

3 Liao, Y., Smyth, G. K. & Shi, W. featureCounts: an efficient general purpose program for assigning sequence reads to genomic features. *Bioinformatics* **30**, 923-930, doi:10.1093/bioinformatics/btt656 (2014).

4 Law, C. W., Chen, Y., Shi, W. & Smyth, G. K. voom: Precision weights unlock linear model analysis tools for RNA-seq read counts. *Genome biology* **15**, R29, doi:10.1186/gb-2014-15-2-r29 (2014).

5 Su, S. *et al.* Glimma: interactive graphics for gene expression analysis. *Bioinformatics* **33**, 2050-2052, doi:10.1093/bioinformatics/btx094 (2017).

6 Pidsley, R. *et al.* A data-driven approach to preprocessing Illumina 450K methylation array data. *BMC Genomics* **14**, 293, doi:10.1186/1471-2164-14-293 (2013).

7 Aryee, M. J. *et al.* Minfi: a flexible and comprehensive Bioconductor package for the analysis of Infinium DNA methylation microarrays. *Bioinformatics* **30**, 1363-1369, doi:10.1093/bioinformatics/btu049 (2014).

8 Ritchie, M. E. *et al.* limma powers differential expression analyses for RNA-sequencing and microarray studies. *Nucleic Acids Res* **43**, e47, doi:10.1093/nar/gkv007 (2015).

9 Skrtic, M. *et al.* Inhibition of mitochondrial translation as a therapeutic strategy for human acute myeloid leukemia. *Cancer Cell* **20**, 674-688, doi:10.1016/j.ccr.2011.10.015 (2011).

10 Janssen, A. J. *et al.* Spectrophotometric assay for complex I of the respiratory chain in tissue samples and cultured fibroblasts. *Clin Chem* **53**, 729-734, doi:10.1373/clinchem.2006.078873 (2007).

11 Jung, C., Higgins, C. M. & Xu, Z. Measuring the quantity and activity of mitochondrial electron transport chain complexes in tissues of central nervous system using blue native polyacrylamide gel electrophoresis. *Anal Biochem* **286**, 214-223, doi:10.1006/abio.2000.4813 (2000).

12 Birch-Machin, M. A. *et al.* Fatal lactic acidosis in infancy with a defect of complex III of the respiratory chain. *Pediatr Res* **25**, 553-559, doi:10.1203/00006450-198905000-00025 (1989).

13 Krahenbuhl, S., Talos, C., Wiesmann, U. & Hoppel, C. L. Development and evaluation of a spectrophotometric assay for complex III in isolated mitochondria, tissues and fibroblasts from rats and humans. *Clin Chim Acta* **230**, 177-187, doi:10.1016/0009-8981(94)90270-4 (1994).

14 Trounce, I. A., Kim, Y. L., Jun, A. S. & Wallace, D. C. Assessment of mitochondrial oxidative phosphorylation in patient muscle biopsies, lymphoblasts, and transmitochondrial cell lines. *Methods Enzymol* **264**, 484-509, doi:10.1016/s0076-6879(96)64044-0 (1996).

15 Brown, M. D., Torroni, A., Shoffner, J. M. & Wallace, D. C. Mitochondrial tRNA(Thr) mutations and lethal infantile mitochondrial myopathy. *Am J Hum Genet* **51**, 446-447 (1992).
16 Wu, H., Southam, A. D., Hines, A. & Viant, M. R. High-throughput tissue extraction protocol for NMR- and MS-based metabolomics. *Anal Biochem* **372**, 204-212, doi:10.1016/j.ab.2007.10.002 (2008).
17 Di Guida, R. *et al.* Non-targeted UHPLC-MS metabolomic data processing methods: a comparative investigation of normalisation, missing value imputation, transformation and scaling. *Metabolomics* **12**, 93, doi:10.1007/s11306-016-1030-9 (2016).
18 Robinson, M. D. & Oshlack, A. A scaling normalization method for differential expression analysis of RNA-seq data. *Genome Biol* **11**, R25, doi:10.1186/gb-2010-11-3-r25 (2010).
19 Kolde, R., Laur, S., Adler, P. & Vilo, J. Robust rank aggregation for gene list integration and meta-analysis. *Bioinformatics* **28**, 573-580, doi:10.1093/bioinformatics/btr709 (2012).
20 Siegel, M. B. *et al.* Small molecule inhibitor screen identifies synergistic activity of the bromodomain inhibitor CPI203 and bortezomib in drug resistant myeloma. *Oncotarget* **6**, 18921-18932, doi:10.18632/oncotarget.4214 (2015).

# Suppelementary Figure 1

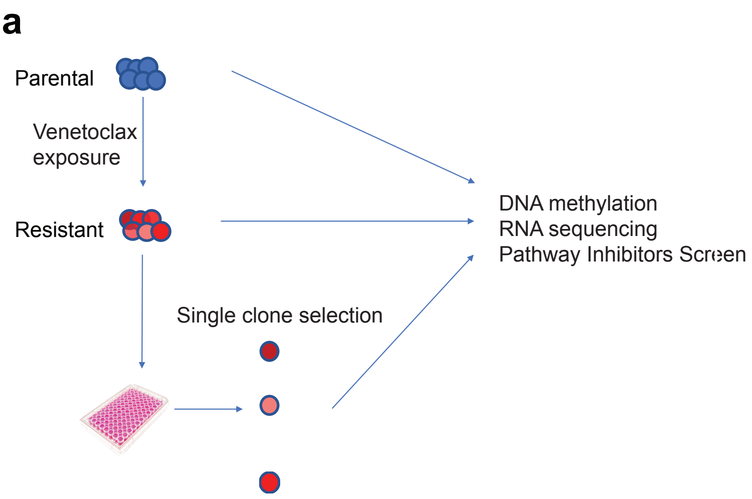


| Parental | VEN-Resistant | Population or Clone |
|---|---|---|
| MV-4-11 | MV-4-11 VEN-RE | Population |
| MOLM-13 | MOLM-13 VEN-RE | Population |
| | MOLM-13 S2 | Clone |
| | MOLM-13 S5 | Clone |
| | MOLM-13 S6 | Clone |
| OCI-AML2 | OCI-AML2 VEN-RE | Population |
| | OCI-AML2 S1 | Clone |
| | OCI-AML2 S2 | Clone |
| | OCI-AML2 S8 | Clone |

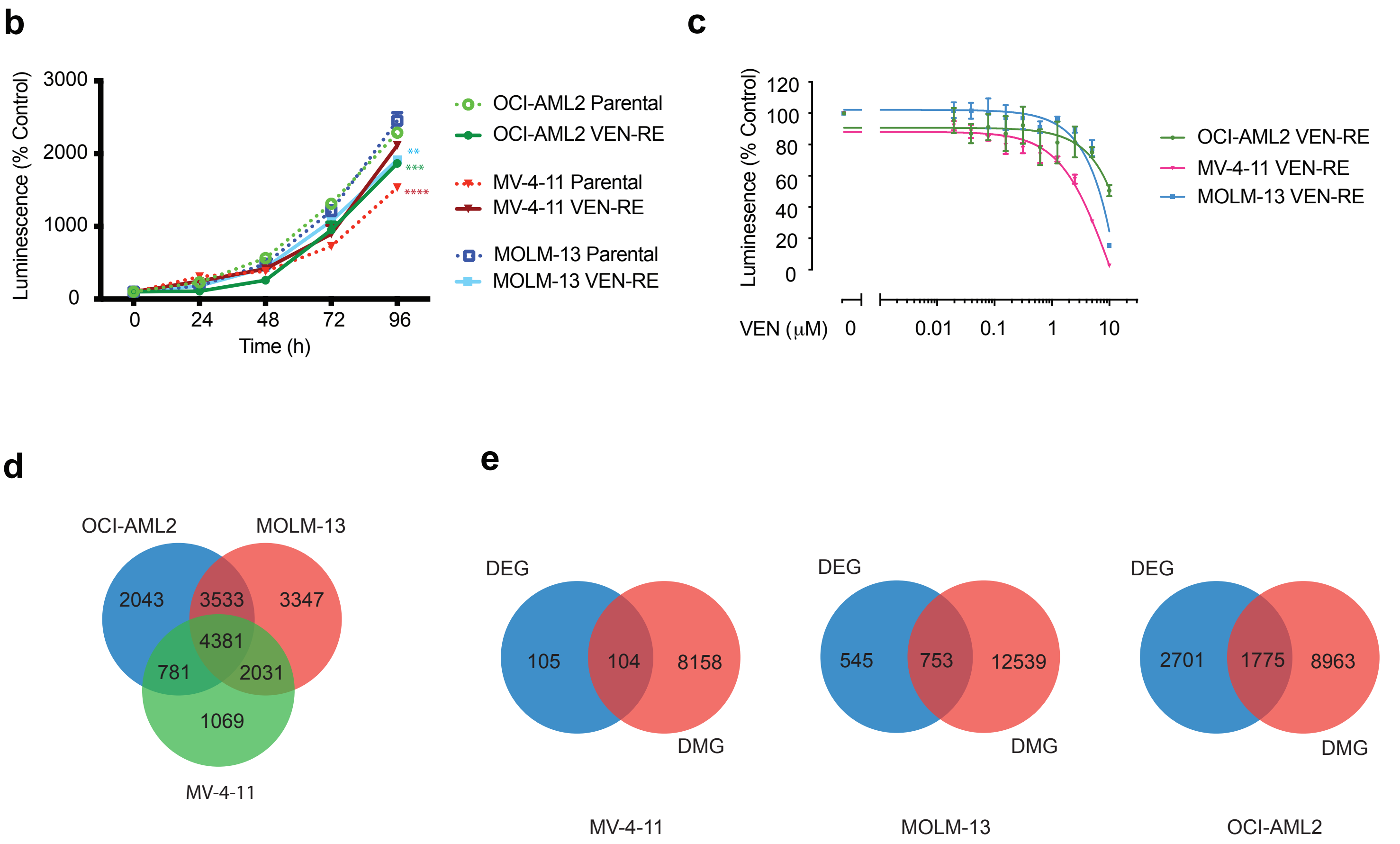


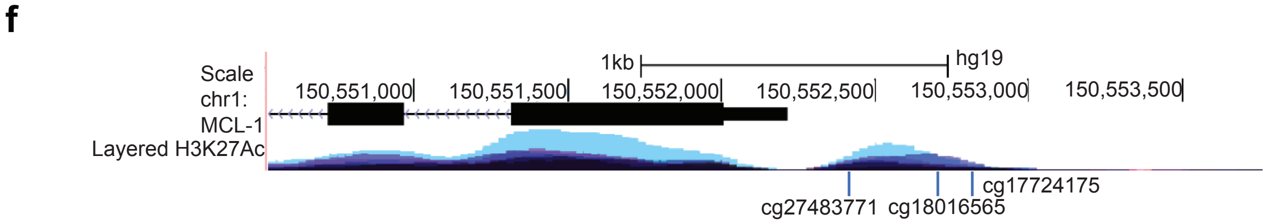


| Probe # | MOLM-13 VEN-RE | OCI-AML2 VEN-RE |
|---|---|---|
| cg17724175 | 15% | NS |
| cg18016565 | 3% | 15% |
| cg27483771 | 16% | NS |

**Supplementary Figure 1. Venetoclax-resistant (VEN-RE) cells show cell-line-specific changes in DNA methylation and RNA sequences.**

(a) Scheme of VEN resistance induction and single-cell clone selection, and list of established VEN-RE cell lines (populations and single-cell clones). (b) Growth curves of the paired parental (dotted lines) and VEN-RE (solid lines) cells. ** $P < 0.005$, *** $P < 0.0005$, **** $P < 0.0001$. (c) VEN-RE cells were washed out of VEN for 48 h before being treated with 0-10 μM VEN for 48 h. Cell viability was determined by CellTiter-Glo assay. (d) Overlap in 3 VEN-RE cell lines of differentially methylated regions (DMR) with at least 20% change in DNA methylation within 2kb of their transcription start site (TSS) (false discovery rate [FDR] 0.1). (e) Overlap of differentially expressed genes (DEG, blue, FDR 0.05) and DMR (red, FDR 0.1) in each pair of cells. (f) Decreased methylation of MCL-1 promotor in VEN-RE MOLM-13 and OCI-AML2 cells. NS: not significant.

## Supplementary Figure 2

**a**

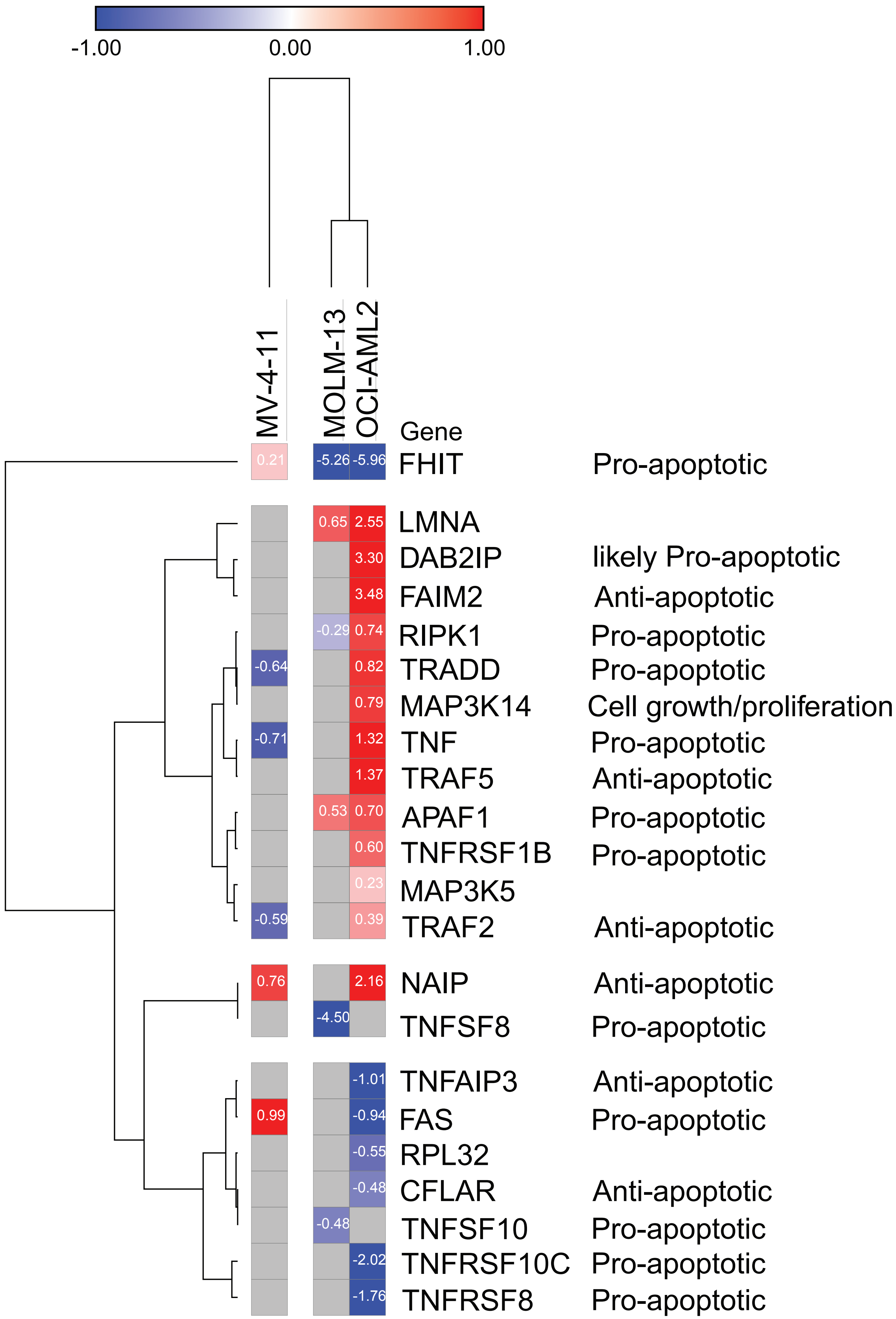

# Supplementary Figure 2

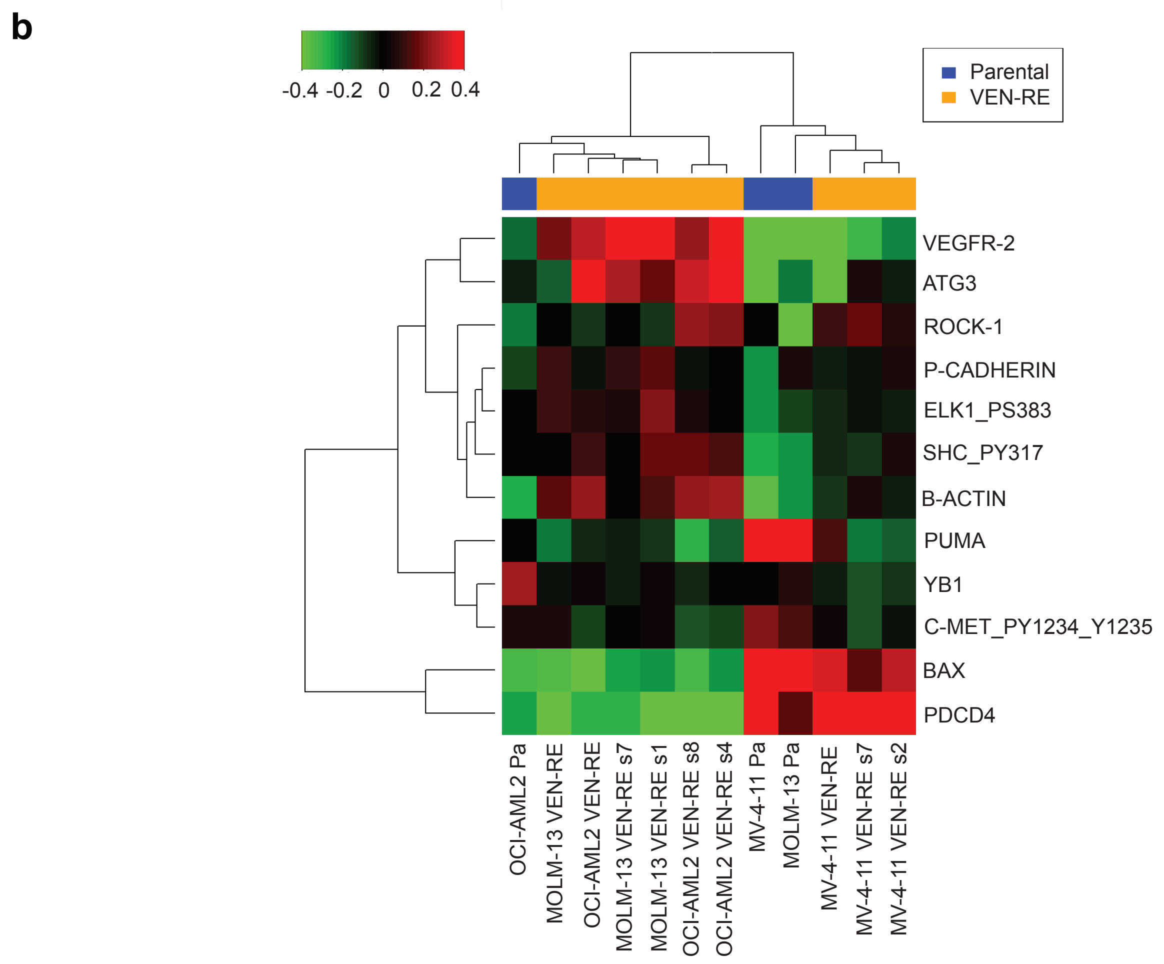


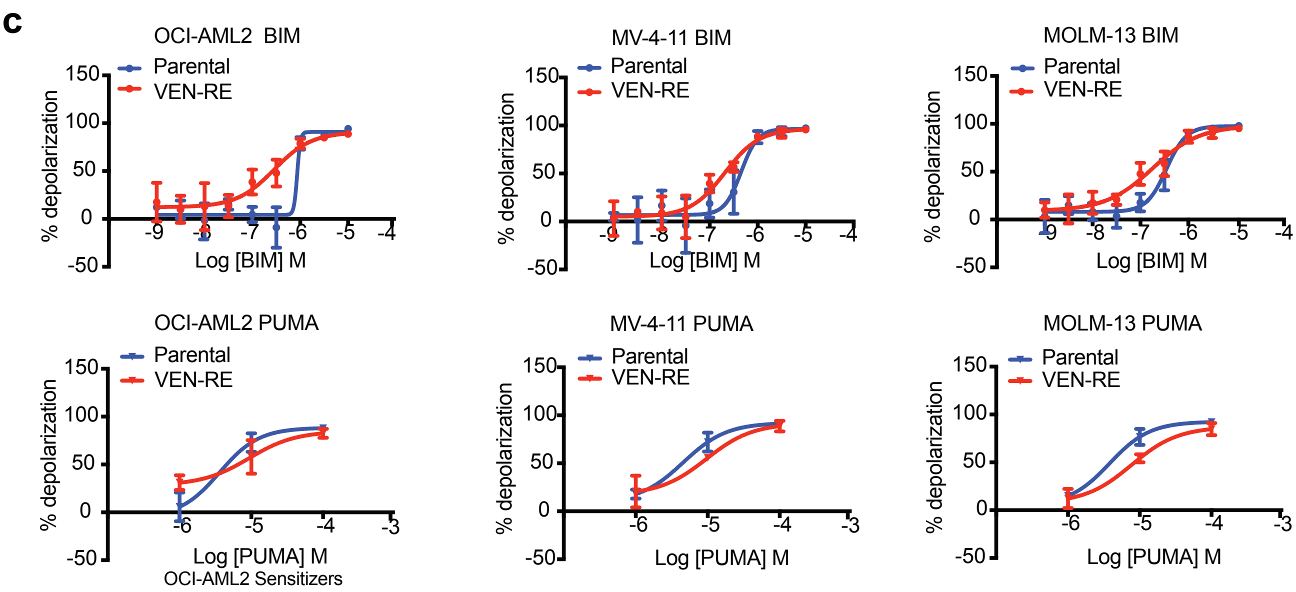


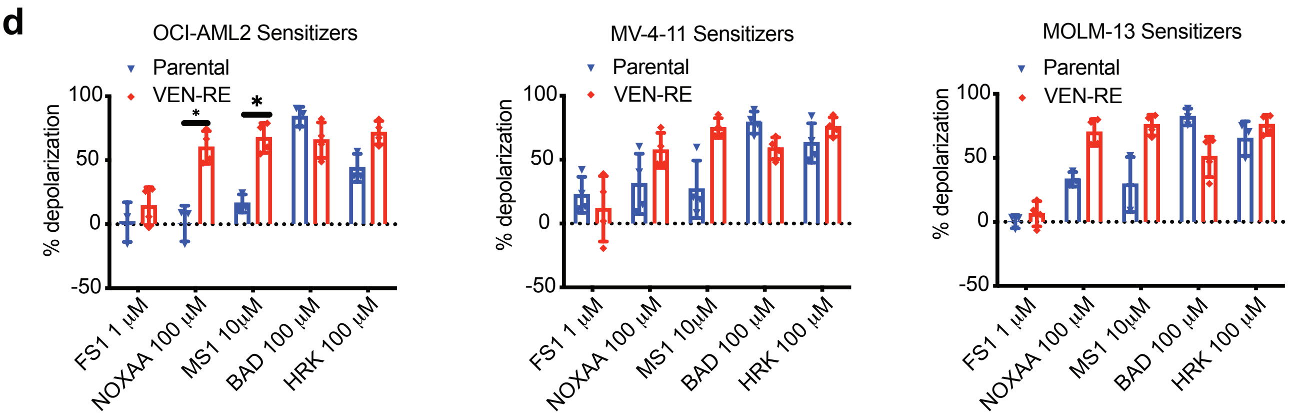

**Supplementary Figure 2. VEN-RE cells maintain sensitivity to MCL-1 inhibition.**

(a) TNF/TRAIL death receptor pathway genes identified by RNA sequencing. FDR ≤ 0.2. Red indicates genes with higher expression, and blue indicates genes with lower expression in VEN-RE cell lines. The numbers in the boxes are expression levels in VEN-RE lines normalized to those in parental lines. (b) RPPA analysis of the proteins differentially expressed in parental and VEN-RE cells. (c) Results of BH3 profiling assays showing mitochondrial depolarization of parental and VEN-RE cells in the presence of the peptides BIM and PUMA. (d) Results of BH3 profiling assays showing mitochondrial depolarization of parental and VEN-RE cells in the presence of the indicated peptides. *$P < 0.05$

## Supplementary Figure 3

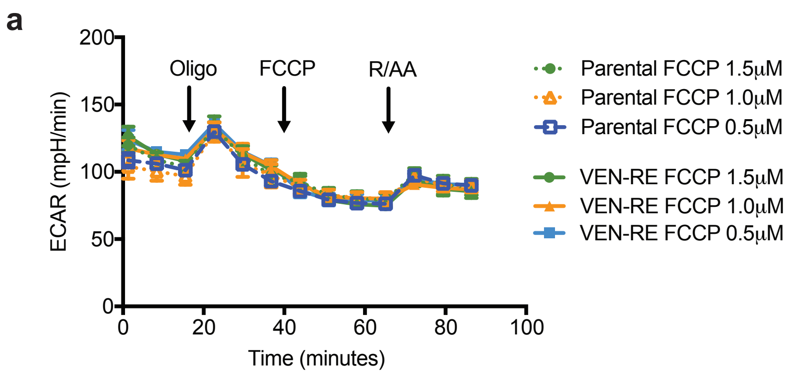


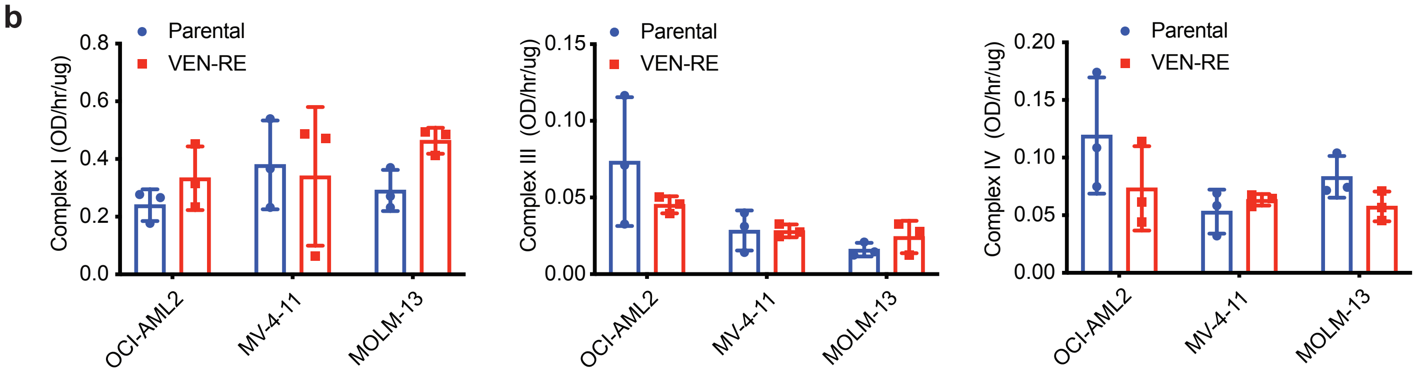

**Supplementary Figure 3**

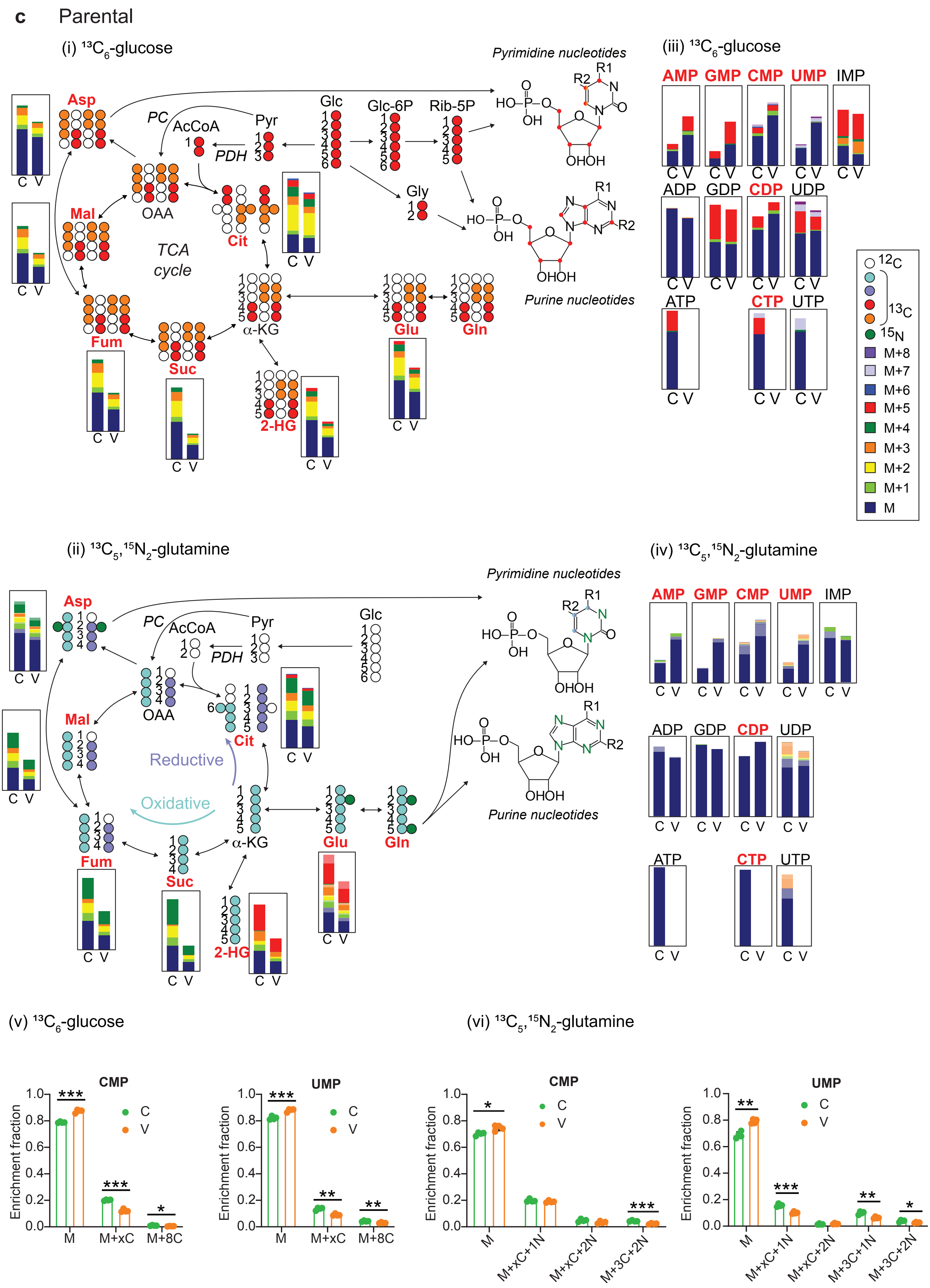

## Supplementary Figure 3

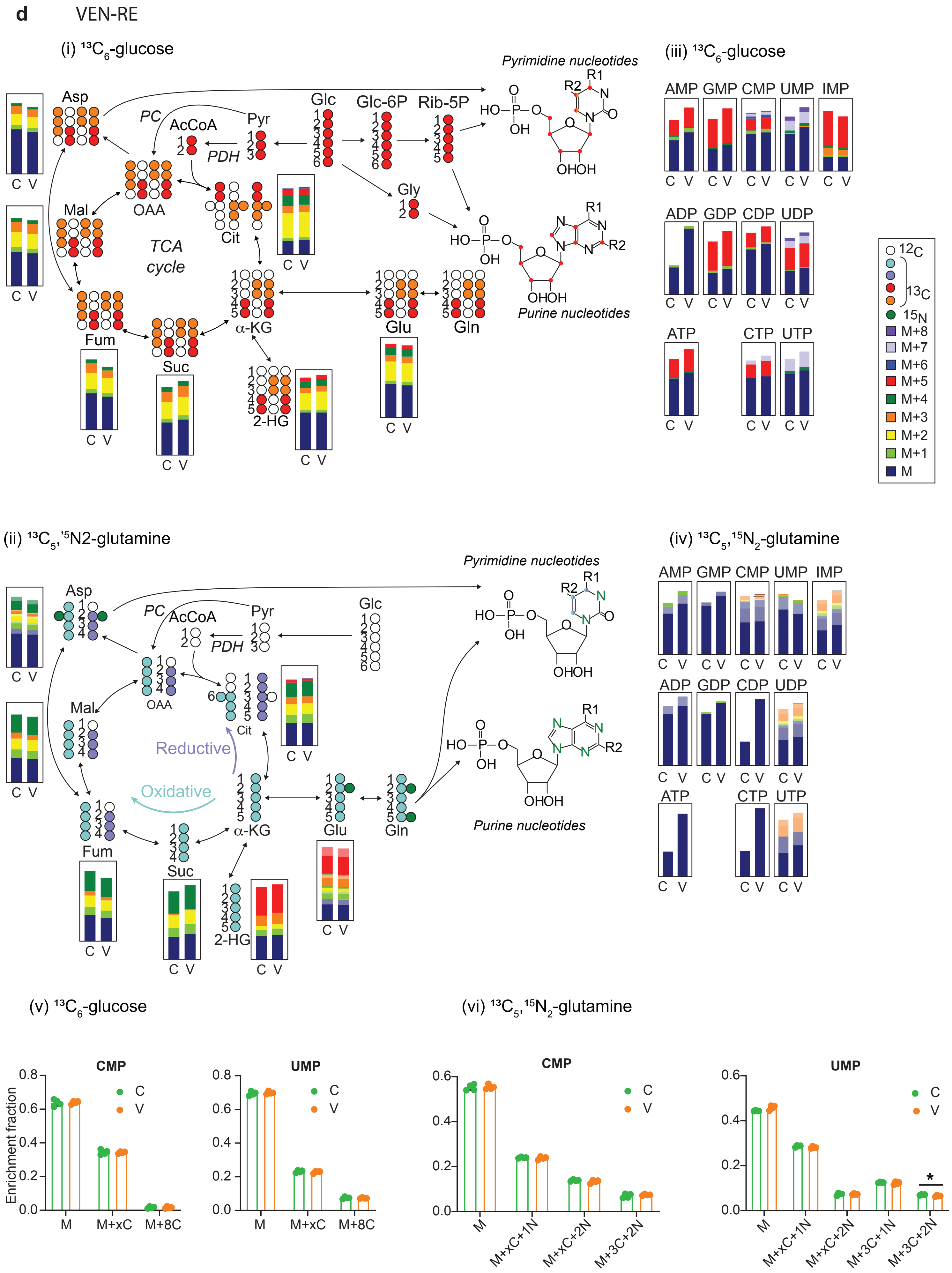

**Supplementary Figure 3**

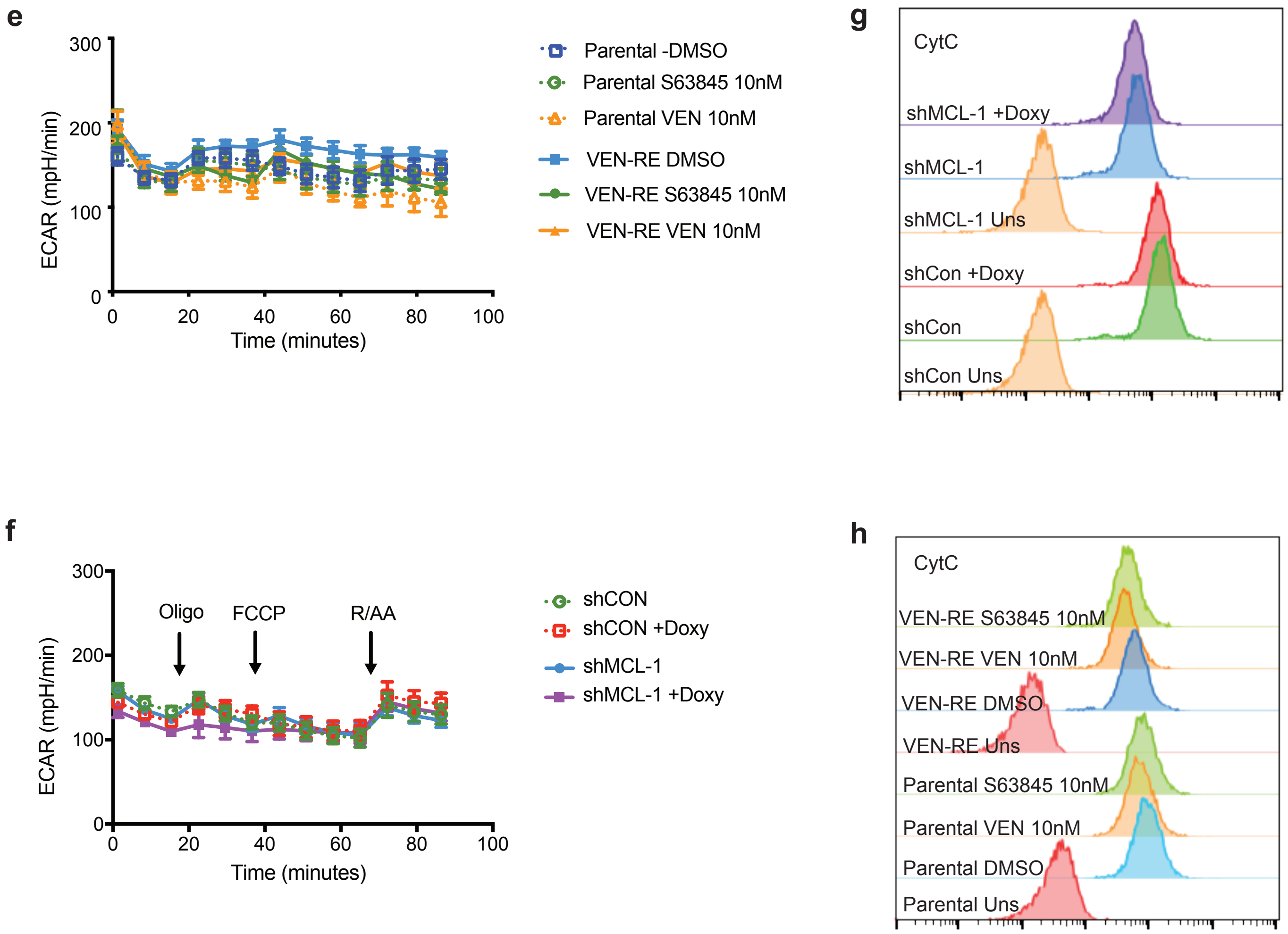

**Supplementary Figure 3. VEN-RE cells exhibit similar ECAR as parental cells.**

(a) Results of Seahorse assays showing extracellular acidification rate (ECAR) with different concentrations of FCCP in parental (dotted lines) and VEN-RE (solid lines) MOLM-13 cells. (b) Electron transport chain complex I, III, and IV activity in parental and VEN-RE cell lines. (c, d) Metabolic modulation induced in parental (c) and VEN-RE (d) MOLM-13 cells following 6 h DMSO control (label as C) or 30 nM VEN treatment (label as V). Relative intensity of isotopically enriched TCA cycle intermediates and nucleotides following labeling with $^{13}C_6$-glucose (i and iii) and $^{13}C_5$,$^{15}N_2$-glutamine (ii and iv) are shown in the bar plots. The color coding shown in the legend refers to enrichment with $^{13}C$. Whenever glutamine-derived $^{15}N$ enrichment is present, those contributions are shown adjacent to the corresponding carbon enrichment level using lighter shades. The metabolites whose total pool levels are significantly different following treatment are bolded and highlighted in red ($P$<0.05). The enrichment fractions of selected nucleoside monophosphates in untreated and VEN-treated parental cells following labeling with $^{13}C_6$-glucose (v) and $^{13}C_5$,$^{15}N_2$-glutamine (vi) show a decreased rate of enrichment (for clarity selected fractions are enlarged in the inserts; *: $P$<0.05, **: $P$<0.005, ***: $P$<0.0005). (e) Results of Seahorse assays showing ECAR in parental and VEN-RE MOLM-13 cells treated with indicted drugs for 1 h. (f) Results of Seahorse assays showing ECAR in VEN-RE MOLM-13 cells transfected with shCON (dotted lines) or shMCL-1 (solid lines) that were induced with or without 100 ng/mL doxycycline for 6 h. (g) Cytochrome C median fluorescence intensity (MFI) by flow cytometry in parental and VEN-RE MOLM-13 cells treated with indicted drugs for 1 h. (h) Cytochrome C MFI by flow cytometry in VEN-RE MOLM-13 cells transfected with a control shRNA (shCON) or a shRNA targeting MCL-1 (shMCL-1) that were induced with or without 100 ng/mL doxycycline for 6 hr.

# Supplementary Figure 4

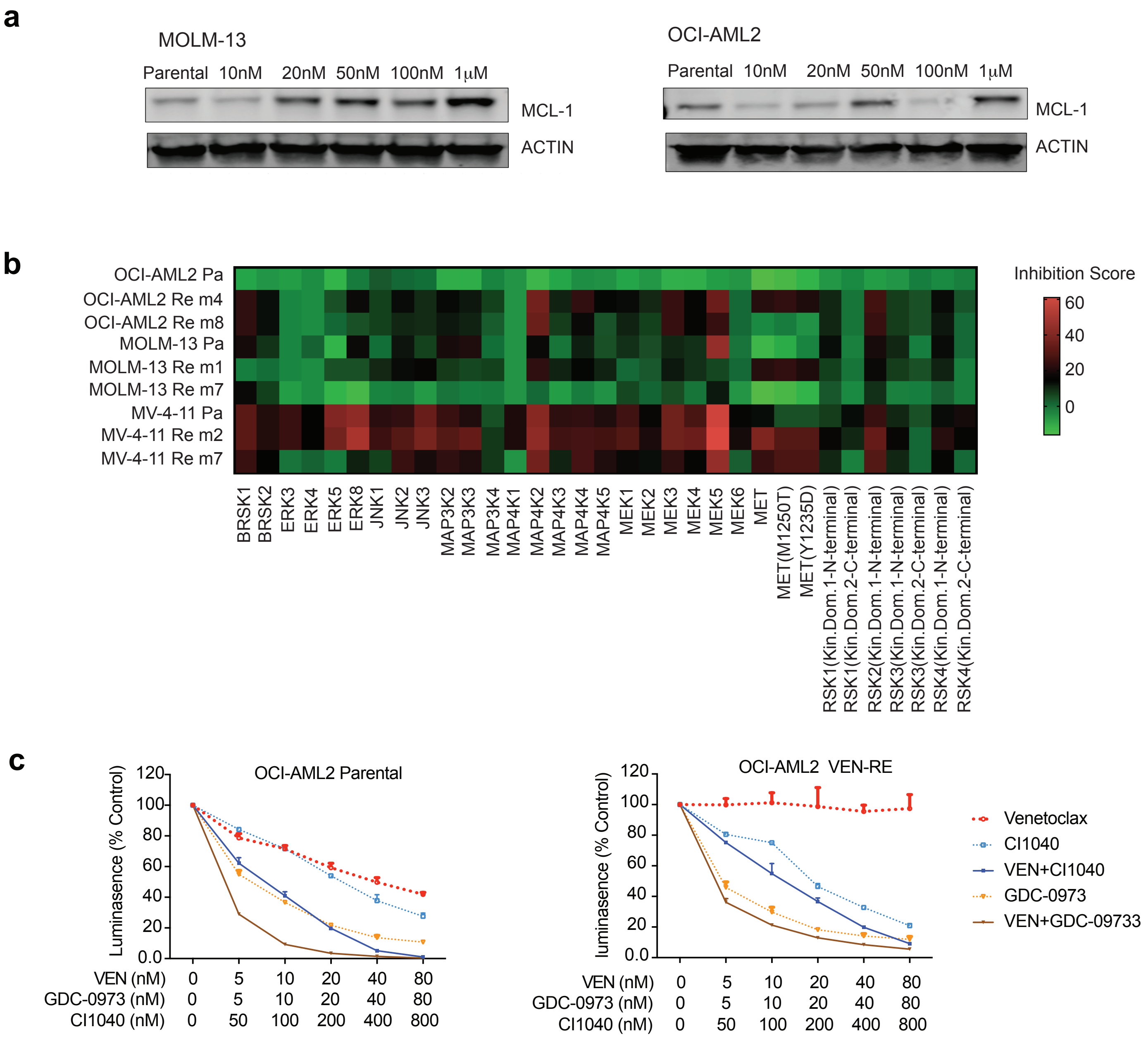

# Supplementary Figure 4

**d**

| MV-4-11 | NAME | NES | FDR q_val |
|---|---|---|---|
| | HALLMARK_KRAS_SIGNALING_UP | 1.93 | 7.50E-04 |
| | HALLMARK_KRAS_SIGNALING_DN | 1.59 | 0.014 |

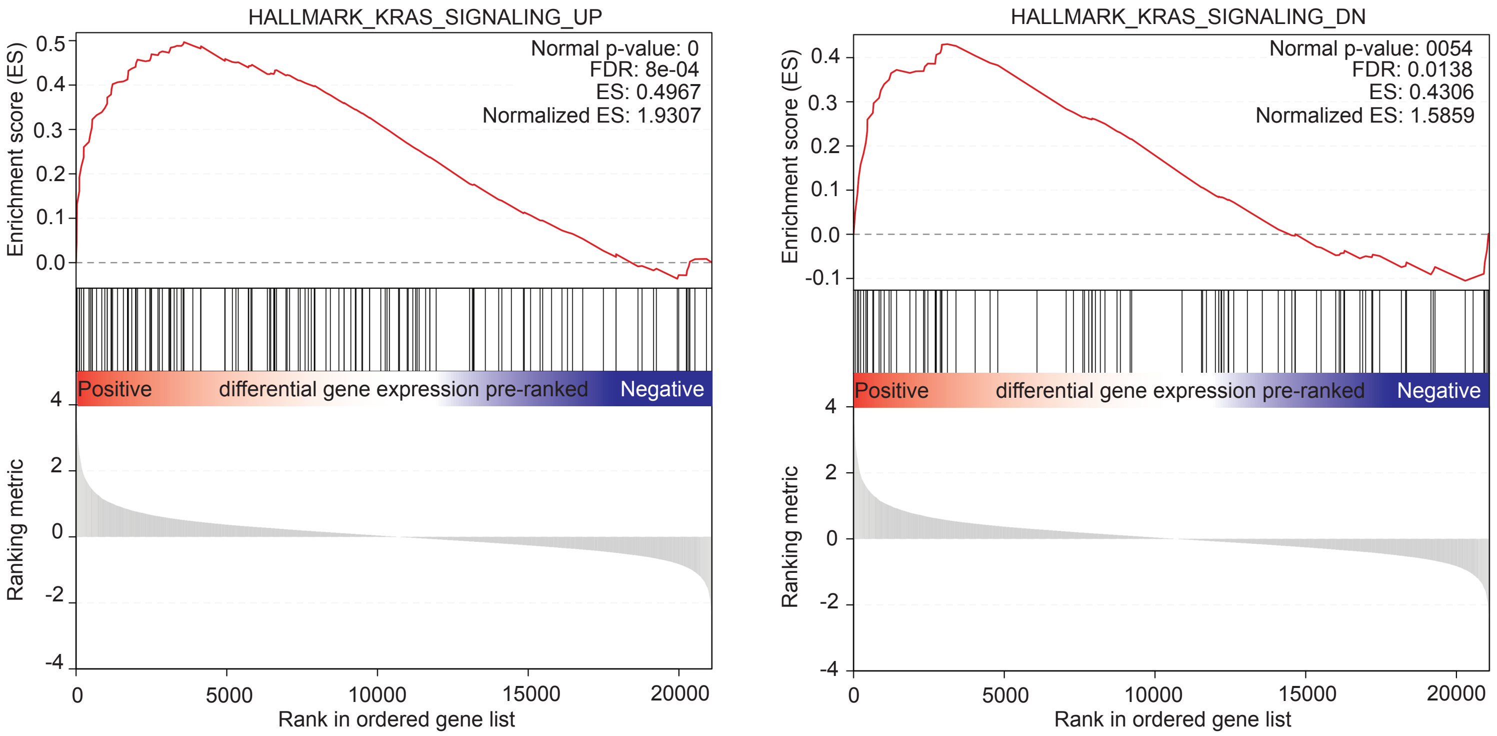


**e**

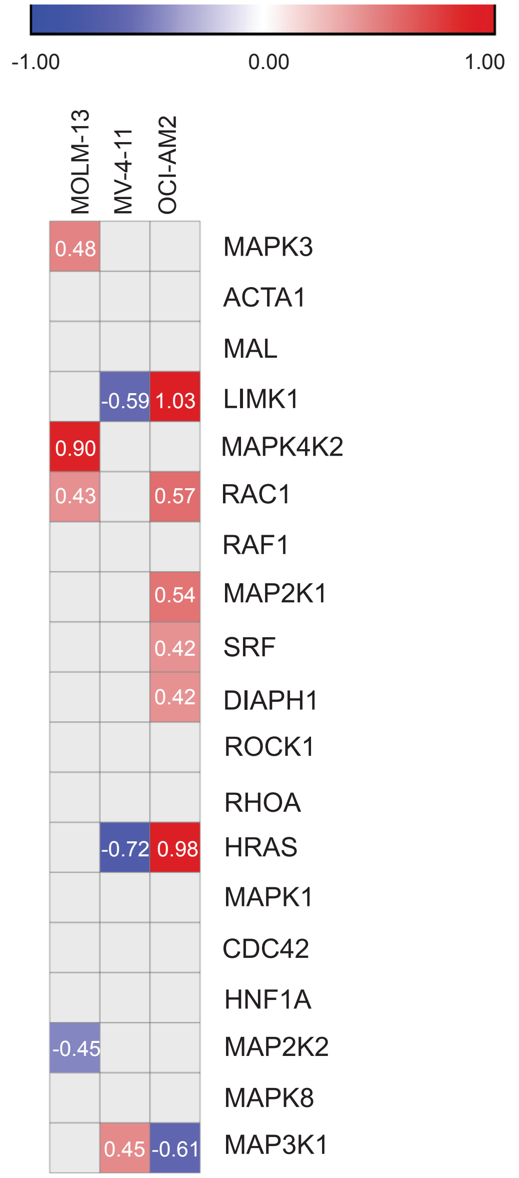

**Supplementary Figure 4. MAPK activation in VEN-RE cells.**

(a) Western blots showing MCL-1 expression in OCI-AML2 and MOLM-13 cells, which have intermediate VEN resistance. (b) Inhibition score of MAPK pathway from inhibitor-library screening. (c) Parental and VEN-RE OCI-AML2 cells were treated with the indicated inhibitors for 48 h, then cell viability was determined by CellTiter-Glo assay. Luminescence reads were normalized to those of DMSO-treated control cells. Data are mean ± SD. (d) Gene set enrichment analysis of RNA sequencing data showing downregulated (top) and upregulated (bottom) genes involved in KRAS signaling in VEN-RE compared to parental MV-4-11 cells. (e) Specific genes in RAS signaling pathway in VEN-RE compared to parental AML cells.

## Supplementary Figure 5

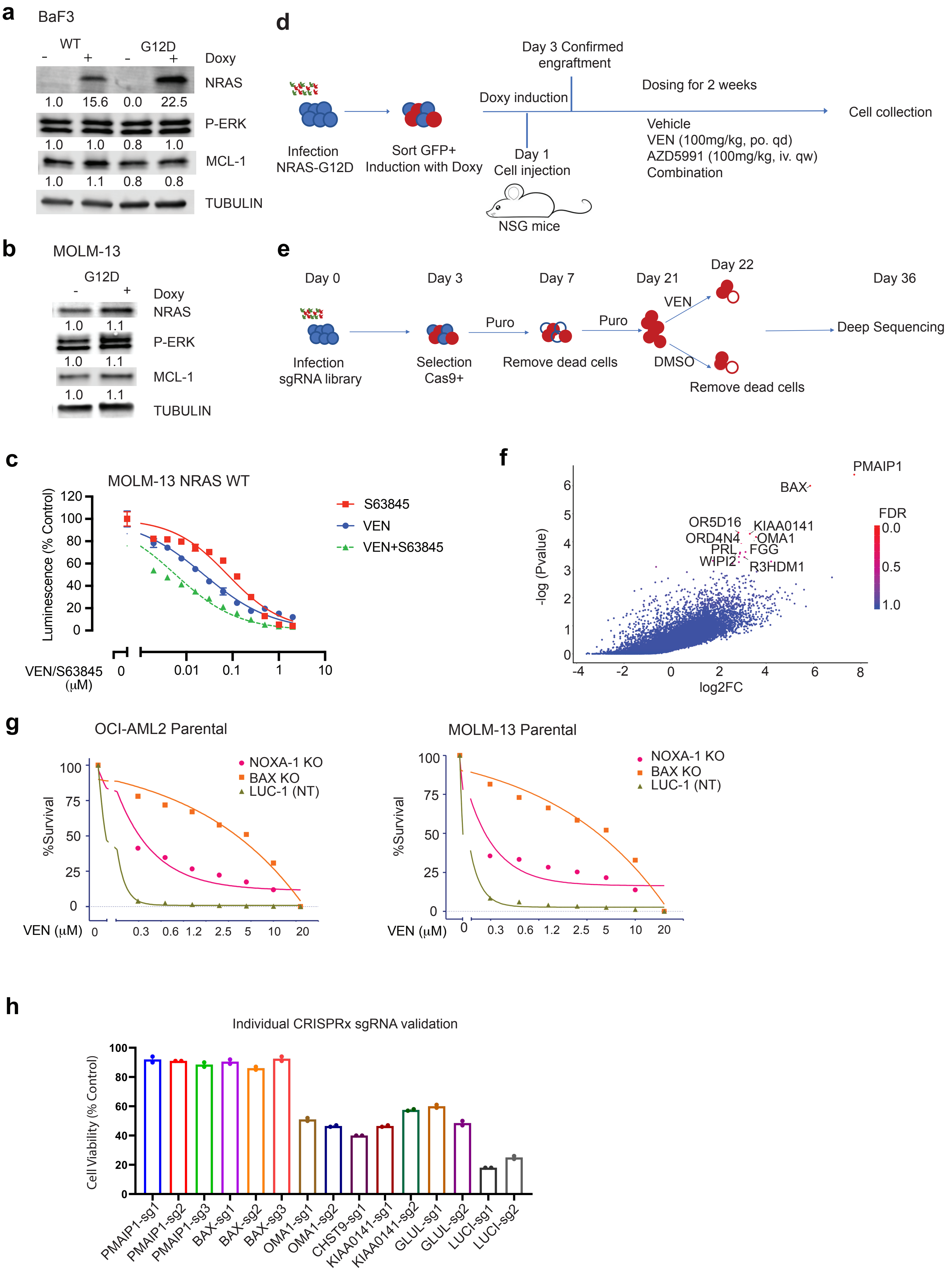

**Supplementary Figure 5. Loss of function of BAX and NOXA and gain of function of adherence-related proteins function contributes to VEN resistance.**

(a) Protein expression by western blot (WB) of BaF3 cells expressing NRAS-WT or NRAS-G12D with or without Doxycycline (1ug/ml) induction for 72 h. (b) Protein expression by western blot (WB) in MOLM-13 cells expressing NARS-G12D with or without Doxycycline (1ug/ml) induction for 72h. (c) MOLM-13 cells expressing NRAS-WT were treated with indicated inhibitor for 24h, then cell viability was determined by CellTiter-Glo assay. Luminescence reads were normalized to those of DMSO-treated control cells. Data are mean ± SD. (d) Schema of MOLM-13 NRAS-G12D *in vivo* experiment. (e) Body weight of experimental mice. (f) Leukemia burden by hCD45/ mCD45 flow cytometry in organs of experimental mice organs. (g) Schema of CRISPR knock-out screening. (h) Results of CRISPR screening of KO52 cells treated with DMSO or 10 μM VEN for 5 days. The surviving cells were deep-sequenced, and genes were plotted by significance. The color scale indicates the false discovery rate (FDR). (i, j) Results of cell viability assays in OCI-AML2 and MOLM-13 cells with CRISPR knockout of NOXA, BAX, and LUC. Cells were treated with 0-20 μM VEN for 24 h. (k) Viability of KO52 cells with CRISPR knockout of genes with significant differences on screening. Cells were treated for 24 h with 5 μM VEN.

**Supplementary Table 1.**

Filtered coding variants for VEN-RE cell line population versus parental cell line.

**Supplementary Table 1. Filtered coding variants for VEN-RE cell line population versus parental cell line**

| | | | | | | | | | RefSeq Genes 105 Interim v1, NCBI | | |
|---|---|---|---|---|---|---|---|---|---|---|---|
| **Cell line** | **Sample** | **Chr:Pos (hg19)** | **Ref/Alt** | **Filter** | **Variant Allele Freq** | **Allelic Depths (AD)** | **Gene Names** | **Sequence Ontology (Combined)** | **Effect (Combined)** | **HGVS c. (Clinically Relevant)** | **Amino acid change** |
| OCIAML2 | VEN-RE_population | 1:32208538 | G/A | PASS | 0.52 | 11,12 | ADGRB2 | missense_variant | Missense | NM_001294335.1:c.1153C>T | p.Arg385Trp |
| OCIAML2 | VEN-RE_population | 3:100617699 | C/A | PASS | 0.76 | 8,25 | ABI3BP | missense_variant | Missense | NM_015429.3:c.389G>T | p.Gly130Val |
| OCIAML2 | VEN-RE_population | 7:156433652 | C/T | PASS | 0.39 | 25,16 | RNF32 | splice_region_variant | Other | NM_030936.3:c.-78+147C>T | |
| OCIAML2 | VEN-RE_population | 9:125330201 | G/C | PASS | 0.35 | 22,12 | OR1L8 | missense_variant | Missense | NM_001004454.1:c.556C>G | p.Leu186Val |
| OCIAML2 | VEN-RE_population | X:18260689 | G/A | PASS | 0.44 | 5,4 | SCML2 | missense_variant | Missense | NM_006089.2:c.1844C>T | p.Pro615Leu |
| OCIAML2 | VEN-RE_population | X:26236040 | G/A | PASS | 1.00 | 0,20 | MAGEB5 | missense_variant | Missense | NM_001271752.1:c.622G>A | p.Ala208Thr |
| OCIAML2 | VEN-RE_population | X:70775066 | T/C | PASS | 1.00 | 0,9 | OGT | missense_variant | Missense | NM_181672.2:c.755T>C | p.Leu252Pro |
| OCIAML2 | VEN-RE_population | X:118217006 | C/A | PASS | 1.00 | 0,10 | KIAA1210 | missense_variant | Missense | NM_020721.1:c.4926G>T | p.Gln1642His |
| MOLM13 | VEN-RE_population | 20:62124543 | C/T | PASS | 0.59 | 11,16 | EEF1A2 | missense_variant | Missense | NM_001958:c.719G>A | p.Arg240His |
| MOLM13 | VEN-RE_population | 7:117267757 | C/A | PASS | 0.45 | 16,13 | CFTR | missense_variant | Missense | NM_000492:c.3650C>A | p.Ala1217Glu |
| MOLM13 | VEN-RE_population | 15:78927903 | C/T | PASS | 0.39 | 19,12 | CHRNB4 | missense_variant | Missense | NM_000750:c.82G>A | p.Glu28Lys |
| MOLM13 | VEN-RE_population | X:70779193 | A/G | PASS | 1.00 | 0,14 | OGT | missense_variant | Missense | NM_181672:c.1679A>G | p.Tyr560Cys |
| MOLM13 | VEN-RE_population | 18:67727126 | T/G | PASS | 0.54 | 11,13 | RTTN | missense_variant | Missense | NM_173630:c.4900A>C | p.Lys1634Gln |
| MOLM13 | VEN-RE_population | 11:89604156 | G/T | PASS | 0.25 | 39,13 | TRIM64B | missense_variant | Missense | NM_001164397:c.983C>A | p.Ala328Asp |
| MOLM13 | VEN-RE_population | 8:133187704 | C/A | PASS | 0.17 | 43,9 | KCNQ3 | missense_variant | Missense | NM_004519:c.929G>T | p.Gly310Val |
| MOLM13 | VEN-RE_population | 8:39782812 | G/T | PASS | 0.15 | 60,11 | IDO1 | missense_variant | Missense | NM_002164:c.778G>T | p.Ala260Ser |
| MOLM13 | VEN-RE_population | 7:24708156 | G/T | PASS | 0.26 | 29,10 | MPP6 | missense_variant | Missense | NM_016447:c.991G>T | p.Ala331Ser |
| MOLM13 | VEN-RE_population | 5:90136695 | C/G | PASS | 0.60 | 19,28 | GPR98 | missense_variant | Missense | NM_032119:c.16912C>G | p.Pro5638Ala |
| MOLM13 | VEN-RE_population | 4:156278625 | T/C | PASS | 0.50 | 15,15 | MAP9 | missense_variant | Missense | NM_001039580:c.1097A>G | p.Asn366Ser |
| MOLM13 | VEN-RE_population | 3:146239822 | G/T | PASS | 0.32 | 15,7 | PLSCR1 | missense_variant | Missense | NM_021105:c.374C>A | p.Thr125Asn |
| MOLM13 | VEN-RE_population | 3:38061776 | G/T | PASS | 0.51 | 18,19 | PLCD1 | missense_variant | Missense | NM_001130964:c.165C>A | p.Ser55Arg |
| MOLM13 | VEN-RE_population | 2:197092923 | A/T | PASS | 0.41 | 16,11 | HECW2 | missense_variant | Missense | NM_020760:c.3820T>A | p.Phe1274Ile |
| MOLM13 | VEN-RE_population | 2:73718478 | C/T | PASS | 0.43 | 12,9 | ALMS1 | missense_variant | Missense | NM_015120:c.9389C>T | p.Ser3130Phe |
| MOLM13 | VEN-RE_population | 1:152284787 | G/T | PASS | 0.71 | 12,30 | FLG | missense_variant | Missense | NM_002016:c.2575C>A | p.Gln859Lys |
| MV411 | VEN-RE_population | 3:36485095 | G/T | PASS | 0.36 | 16,9 | STAC | missense_variant | Missense | NM_003149.2:c.351G>T | p.Lys117Asn |
| MV411 | VEN-RE_population | X:53239923 | C/T | PASS | 0.57 | 12,16 | KDM5C | missense_variant | Missense | NM_004187.3:c.1518G>A | p.Met506Ile |
| MV411 | VEN-RE_population | 19:2339061 | G/C | PASS | 0.26 | 31,11 | SPPL2B | splice_region_variant | Other | NM_152988.2:c.460-7G>C | |
| MV411 | VEN-RE_population | 2:32704640 | C/T | PASS | 0.24 | 19,6 | BIRC6 | stop_gained | LoF | NM_016252.3:c.7363C>T | p.Gln2455Ter |
| MV411 | VEN-RE_population | 21:31802902 | C/A | PASS | 0.26 | 14,5 | KRTAP13-4 | stop_gained | LoF | NM_181600.1:c.309C>A | p.Tyr103Ter |

**Supplementary Table 2.**

Differentially methylated regions between parental and VEN-RE cells.

| | Differentially methylated regions (DMRs) | | | |
|---|---|---|---|---|
| **Cell lines compared** | **FDR[*] ≤ 0.1** | **FDR ≤ 0.1 +Δβ[**] ≥ 20%** | **FDR ≤ 0.1 +Δβ ≥ 20% +2 kb TSS[***]** | **Gene** |
| VEN-RE Pop and Clones vs Parental | 511 761 | 12568 | 4999 | 3423 |
| OCI-AML2 VEN-RE Pop and Clones vs Parental | 480403 | 67470 | 20044 | 10738 |
| MV-4-11 VEN-RE Pop vs Parental | 282849 | 36776 | 14723 | 8262 |
| MOLM-13 VEN-RE Pop and Clones vs Parental | 547174 | 101581 | 36015 | 13292 |

* FDR: false discovery rate.

**Δβ: mean methylation delta value

***TSS: transcription start site.

**Supplementary Table 3.**

Clinical and genomic characteristics of patients who experienced AML relapse on VEN-based trial that used for single- cell sequencing.

| Sample | Time point | Blast % | Days to relapse | Genetics | # Cells sequenced |
|---|---|---|---|---|---|
| 1-7992 | Screen | 82 | | FLT3-ITD, KRAS, NRAS, TP53 | 4616 |
| | Relapse | 28 | 131 | | 3780 |
| 2-4006 | Screen | 17 | | ASLX1, NRAS, NPM1 | 5781 |
| | Relapse | 12 | 294 | | 8837 |

**Supplementary Table 4.**

Clinical and genomic characteristics of patients who experienced AML relapse (3-3570 and 4-3072) or were primary refractory (5-0716 and 6-9696) on VEN-based trial that used for IHC and WB.

| Sample | Time point | Blast % | Days to relapse | Genetics |
|---|---|---|---|---|
| 3-3570 | Screen | 25 | | IDH1, NPM1, NRAS, WT1, CEBPA, DNMT3 |
| | Relapse | 73 | 117 | |
| 4-3072 | Screen | 18 | | DNMT3A, NPM1, NRAS, ASXL1, IGH, TCRG |
| | Relapse | 55 | 302 | |
| 5-0716 | Screen | 72 | | KRAS, NRAS, RUNX1, SF3B1, HNRNPK, IKZF1, NF1 |
| | Refractory | 74 | 57 | |
| 6-9696 | Screen | 38 | | KRAS, NF1, NRAS, NF1, PTPN11, TP53x2 |
| | Refractory | 34 | 31 | |
